# Can Complex 3D Models Effectively Replace 2D and Animal Models to Investigate the Microbe-Tumor-Immune Axis in Pancreatic Cancer Studies?

**DOI:** 10.3390/nu18132113

**Published:** 2026-06-28

**Authors:** Fathima Zahraa Ozeer, Jemila Caplan Kester

**Affiliations:** School of Science, Faculty of Health and Environmental Sciences, Auckland University of Technology, Auckland 1010, New Zealand; qnh6203@autuni.ac.nz

**Keywords:** pancreatic cancer, immune checkpoint inhibitor, organoids, spheroids, microbiomes

## Abstract

The tumor microbiome has been implicated in pancreatic ductal adenocarcinoma (PDAC)’s poor response to treatment, demanding new methods for understanding host-microbe interactions in therapy. Traditional 2D systems, while widely used, fail to adequately recapitulate human PDAC due to insufficient representation of structural, immunological and stromal components. Differences in cancer-specific microbiomes, microbe-immune interactions, and the unique physiological and immunosuppressive features unique to PDAC have hindered the clinical translation of immune therapies. Reproducible 3D culture systems that integrate the human microbe-tumor-immune (MTI) axis represent a promising avenue for treatment research, yet they remain underexplored in PDAC. In this narrative review, we discuss the key microbial determinants of therapy resistance, explore the current 3D multicellular modeling approaches in other cancer types, and provide a path forward for similar integrative translational models in PDAC.

## 1. Introduction

Pancreatic ductal adenocarcinoma (PDAC) is a solid tumor accounting for over 90% of pancreatic cancer cases [1]. It remains one of the most lethal malignancies with a five-year survival rate of just 13% (United States 2013–2019, [2]). Symptoms of PDAC are usually non-specific, and there is no PDAC screening test, leading to late diagnosis [3,4]. At this advanced stage, the tumor has metastasized beyond the pancreas, making surgery ineffective. For operable tumors, surgery is often unsuccessful in PDAC, with high recurrence rates post-resection likely due to increased venous invasion [5]. Both radiotherapy and first-line chemotherapy show limited treatment response [6] due to physicochemical and immune properties, discussed in Section 2. A promising alternative therapeutic strategy is the use of immunotherapies, which involves bolstering the host’s immune system towards recognizing cancer cells as a threat. Immune checkpoint inhibitor (ICI) therapy is a type of immunotherapy that blocks T-cell and cancer cell receptors, such as programmed cell death receptor (PD-1) and its ligand (PD-L1), responsible for bypassing cancer cell recognition and apoptosis.

Although ICI therapy has resulted in improved overall survival (OS) rates in other solid tumors like melanoma and lung cancers, pancreatic cancer has shown limited response to both monotherapies involving single target receptors and combination treatments with chemotherapy [7,8]. Reduced effectiveness of immunotherapies in PDAC has a multifactorial etiology, caused by genetic, tumor-intrinsic, and immune contributions: checkpoint inhibitor monotherapies are approved for solid tumors with mismatch repair mutations, but over 99% of PDAC tumors do not exhibit this mutation [9]; PDAC tumor cells lack sufficient intrinsic antigenicity and antigen presentation to elicit an effective immune response while they simultaneously suppress immune cells directly [10]; and PDAC tumors are characterized by a fibrotic, or desmoplastic, stroma which forms a mechanical barrier and an immune suppressive tumor microenvironment (TME), hindering immune cell infiltration and function further. This highlights the urgent need to alter this tumor promoting immune microenvironment and to employ alternative immune-modulating approaches beyond conventional treatments [7,11].

Increasing evidence shows that both commensal and tumor-associated microbiota strongly associate with tumor initiation and progression, immune modulation, and response to treatment of PDAC [6,12]. However, interpreting causality from these associations should be done cautiously until direct links can be established. Therefore, investigating the mechanistic influence of microbes on the immune and therapeutic landscape of PDAC is critical. A primary hurdle for understanding these mechanistic interactions has been a lack of physiologically relevant models.

Two-dimensional (2D) culture models are widely used due to their affordability and reproducibility; however, their simplistic monolayer architecture fails to capture the phenotypic and structural complexity of tumors. Although animal models are frequently utilized in PDAC research, their translational relevance to human systems is often limited due to physiological differences, lack of immune and stromal complexity, and high associated costs [13]. In contrast to 2D models, three-dimensional (3D) models more closely recapitulate tumor structures and stromal composition, while also being cost-effective compared to in vivo systems. Additionally, 3D systems can be flexibly cultured with other components, such as drugs, immune cells, stromal cells [13], and microbes to investigate complex cross-interactions [14]. 3D models, though, are limited in their relative expense over standard 2D models, especially due to the requirement for human growth factors and cytokines and the extensive troubleshooting needed to validate them. For large-scale studies, these models typically lack the scalability afforded by 2D models. Additionally, 3D models may not fully recapitulate in vivo dynamics either.

Despite increasing evidence that the microbiome profoundly shapes the immune landscape of tumors, thus influencing responses to immunotherapy, integrative 3D culture models incorporating microbial and immune components remain exceedingly limited in PDAC. Organoid technology involving the immune-microbe axis has been widely adopted in colorectal (CRC) and gastric cancer research; however, similar integration in PDAC organoids has proved challenging [12], likely due to the tumor’s unique desmoplastic architecture and complex TME. A significant gap persists in the development of reproducible multi-culture, or heterotypic, 3D systems capable of recapitulating PDAC’s intricate stromal composition, structural heterogeneity, and dynamic immune–microbial interplay.

In this review, we summarize the microbial influences on immune modulation, oncogenesis, and therapy resistance in PDAC, as well as the translational capability of current preclinical models. Collectively, this emphasizes the pressing necessity to develop integrative, clinically translational models for future PDAC research to improve therapeutic outcomes.

### Methods

We reviewed the MTI literature to summarize what is known in PDAC models and identify current barriers. PubMed, Google Scholar, and Medline were searched, and only peer-reviewed papers originally in English were included. Searches were conducted through June 2026 for all relevant articles. Papers were screened through and selected based on content Keywords used were “microbes”, “bacteria”, “cancer”, “PDAC”, “immune modulation”, “immune response”, “immune infiltration”, “cytotoxic”, “three-dimensional systems”, “organoids”, “spheroids”, “heterotypic”, “multicellular”, “recapitulate” and “in vivo PDAC” and combinations of the above. Review papers were used as a guided source of information for obtaining original journal articles. Where claims could not be validated, the article was excluded. Targeted ad hoc searches were also conducted using Google for specific keywords, with the same rigorous exclusion criteria applied as above.

## 2. The PDAC Tumor Microenvironment

**Figure 1:**
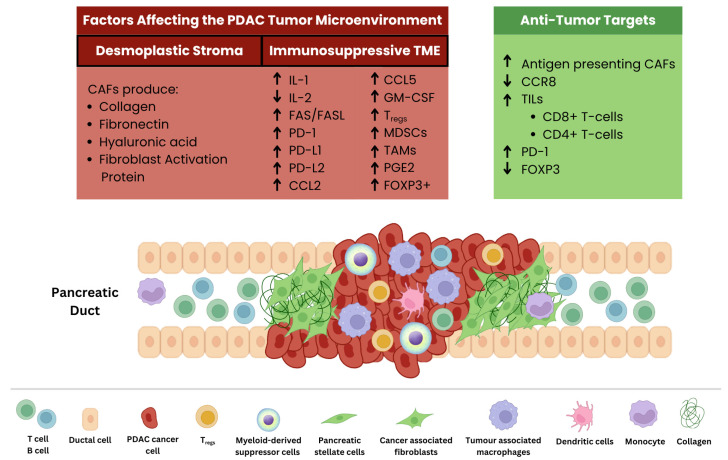
Schematic diagram of the desmoplastic and immunosuppressive TME of an in vivo PDAC tumor. Collagen, fibronectin, and hyaluronic acid produced by the CAFs form a barrier preventing B and T-cells from entering the tumor. CAFs additionally suppress differentiation and promote dysfunction, exhaustion, and death of CD8^+^ T-cells via suppressed IL-2 and upregulation of PD-1, PD-L1, PD-L2, and FAS/FASL, as well as increased production of PGE2. Immunosuppressive cells like T_regs_ and MDSCs hinder T-cell activation and contribute to an immunosuppressive tumor microenvironment through increased CCR8 and *FOXP3* expression. This, in turn, increases CCL5, suppressing anti-tumor activity. Up arrows indicate increased expression, down arrows indicate decreased expression. Abbreviations at the end of the article.

PDAC tumors are situated within a complex system of cellular and molecular components, fibrous matrices, collapsed blood vessels, and an overall uniquely immunosuppressive TME (Figure 1). This TME is critical to recapitulate in any model used to understand immune response within PDAC, and yet the most common 2D model systems lack the complexity to mimic the immunosuppressive nature of PDAC. 3D models can be used to better model this aspect of PDAC, as we discuss in Section 3.

These cellular components of the TME include stromal cells (e.g., pancreatic stellate cells (PSCs) and their activated form cancer-associated fibroblasts (CAFs)), endothelial cells, and immune cells (e.g., macrophages, B-cells, T-cells). Additionally, there is an extracellular matrix (ECM) rich in growth factors and cytokines that supports tumor maintenance and progression. This complex network forms a dense and immunosuppressive stroma that drives disease progression and contributes to poor therapeutic outcomes and low survival rates in PDAC [4,6,8].

Anti-inflammatory cytokines such as interleukin-10 (IL-10), pro-inflammatory cytokines including IL-1, IL-6, IL-17, and tumor necrosis factor-α (TNF-α), and the bidirectional immunomodulatory cytokine transforming growth factor-β (TGF-β) are critically involved in the pathophysiology of PDAC. However, pro-inflammatory cytokines are not always anti-tumor, as the temporal and spatial control of their expression can matter significantly. Immunomodulatory cytokine expression is dynamically regulated by interactions between tumor cells and infiltrating immune cells [15]. “Hot” tumors are defined by a strong presence of active, non-exhausted T-cells within the TME, particularly a high density of tumor-killing CD8^+^ T-cells [16]. On the contrary, PDAC possesses an immunologically “cold” TME, defined by the accumulation of immunosuppressive cells and extracellular factors within the tumor and surrounding stroma (Figure 1). The major classes of immunosuppressive cells include tumor-associated macrophages (TAMs), CAFs, myeloid-derived suppressor cells (MDSCs), and regulatory T-cells (T_regs_) [6,15,17].

TAMs have emerged as a particularly important cell type in many cancers, including PDAC. While in vitro studies classically described macrophages as polarizing to either M1 anti-inflammatory or M2 pro-inflammatory, in vivo work has clearly demonstrated that a more complex system is at play, where both M1 and M2—and possibly an intermediate state termed ‘alternatively activated’—likely contribute to the pro-tumorigenic TME [18,19]. Further studies, especially using MTI models, are needed to fully parse the complexity of TAMs in PDAC.

Stellate cell-derived CAFs, a heterogeneous group of cells responsible for producing ECM components such as collagen, hyaluronic acid and fibronectin, are the main contributors to desmoplasia [3,4]. CAFs produce a cell surface fibroblast activation protein (FAP), which has been associated with oncogenic effects such as angiogenesis, tumorigenesis, metastasis and immunosuppression [3,8].

CAFs can reversibly differentiate into myofibroblastic or immune-mediating subtypes. Myofibroblastic CAFs (myCAFs), triggered by TGF-β, express high levels of α-smooth muscle actin (α-SMA), which is responsible for producing the ECM proteins primarily constituting the surrounding stroma [3,20]. Additionally, a positive correlation between large xenograft tumors and their close proximity to myCAFs indicates that their secretions promote PDAC expansion [21]. The depletion of myCAFs using diphtheria toxin accumulated cytotoxic T-lymphocytes (CTLs) and enhanced PD-L1 therapy [22]. In contrast, genetic deletion of myCAFs resulted in a more aggressive and invasive phenotype of pancreatic tumor cells [3,23,24]. Collectively, these findings suggest that myCAFs are not solely tumor-promoting but also possess a tumor-restraining role. Inflammatory CAFs (iCAFs) are stimulated by IL-1 and encourage proliferative tumorigenic effects like PDAC solid stress through the production of hyaluronan. iCAFs express low levels of α-SMA but high levels of the type I angiotensin II receptor responsible for vasoconstriction and increased blood pressure. The inhibition of this receptor results in decreased intratumoral solid stress, resulting in improved infiltration and drug delivery [20,25]. On the other hand, another CAF subpopulation termed antigen-presenting CAFs expresses major-histocompatibility complex (MHC) class II molecules, which, in the presence of sufficient co-stimulatory molecules to produce an effective response, are capable of presenting antigens to CD4^+^ T-cells, a process important for antitumor immune responses [20,25].

In PDAC, CAFs are major drivers of immune suppression and tumor progression through both contact-dependent and soluble immunosuppressive mechanisms (Figure 1). For instance, by suppressing IL-2 production, CAFs significantly impair the proliferation and differentiation of CD8^+^ T-cell function. Furthermore, the upregulation of cell surface proteins like Fas ligand (FASL) and the PD-1 ligand PD-L2 on CAFs binds to their respective receptors (Fas and PD-1), causing chronic stimulation of T-cells, leading to reduced proliferation, dysfunction and death of CD4^+^ and CD8^+^ T-cells, also known as T-cell exhaustion, ultimately promoting tumor cell survival [26,27]. Furthermore, CAFs produce the soluble factor prostaglandin E2 (PGE2), and the inhibition of PGE2 restored T-cell proliferation partially [27]. This implies that CAFs play a significant role in maintaining the immunosuppressive TME in PDAC. However, the situation may be more complex in clinical settings.

CAFs exhibit a complex and bidirectional role in immune modulation. Interestingly, exposure of CAFs to radiation induces a senescent phenotype, which in turn enhances their tumor-promoting and immunosuppressive functions. This is evidenced by increased surface expression of immune checkpoint and immunoregulatory receptors, as well as ectonucleotidases that convert extracellular ATP into immunosuppressive adenosine, thereby further reinforcing an immunosuppressive TME [24]. Taken together, these data show that CAFs in PDAC are not exclusively tumor-promoting, despite their predominant role in driving tumor progression (Figure 1). Certain CAF subpopulations can support antitumor immunity, while indiscriminate depletion of CAFs may be therapeutically detrimental. This highlights their heterogeneous and context-dependent role in tumorigenesis. Furthermore, radiotherapy may enhance CAF-mediated immunosuppressive remodeling of the TME, thereby contributing to the profound therapy resistance observed in PDAC.

The varying roles of CAFs may also be location-dependent. Within the PDAC tumor, reactive sub-regions house high levels of activated CAFs expressing FAPα and α-SMA, and secreting inflammatory cytokine IL-6, forming the intratumoral TME. These reactive regions are chemo-sensitive and establish an inflammatory TME enriched in T-cells, TAMs, and endothelial cells in close contact with tumor cells. While this is a generally anti-tumorigenic immune profile, reactive sub-regions also exhibited increased markers for T-cell exhaustion (PD-L1) and suppressive T_regs_ (FOXP3). This suggests that immunosuppressive TMEs are encouraged by extensive T-cell activation and exhaustion, collectively enhancing tumorigenesis. Contrastingly, other sub-regions have minimally activated CAFs at lower concentrations expressing high levels of B-cell markers, high collagen composition, and show chemoprotective effects [28].

As part of the overall TME, TAMs and CAFs make up most of the cellular components of the stroma. They are often found in close proximity to one another and jointly contribute towards immunosuppressive desmoplasia [29]. TAMs and CAFs mutually reinforce each other’s immunosuppressive functions through a positive feedback loop within the TME. TAM-derived cytokines such as TGF-β and IL-1β promote CAF differentiation and activation, while CAFs were shown to secrete IL-33, IL-6 and granulocyte–macrophage colony-stimulating factor (GM-CSF), which drive macrophage polarization toward the pro-tumorigenic M2 phenotype in several cancer types [30,31,32]. Together, these bidirectional interactions maintain the immunosuppressive and tumor-supportive PDAC TME, while the substantial heterogeneity between TME subregions likely contributes to differential therapeutic responses, limiting the efficacy of uniform treatment approaches.

MDSCs, another important cell type in the PDAC TME, are recruited to PDAC sites through increased expression of GM-CSF [33] and other factors. Once resident, MDSCs contribute to the immunosuppressive environment through three synergistic mechanisms [34]. First, they produce reactive oxygen species in a STAT3-dependent mechanism [35], leading to natural killer (NK) cell impairment. Second, they produce nitric oxide in a STAT1-dependent mechanism [36], which causes depletion of CD8^+^ T-cells [37]. Thirdly, they produce arginase 1, an enzyme that depletes arginine from the TME [38], leading to T-cell exhaustion. Together, these three mechanisms induce anergy in T and NK cells [39], recruit T_reg_ cells [40], and ultimately reduce ICI efficacy and patient survival [41].

An important component of the cold PDAC tumor is T_regs,_ which assist with immune evasion in TMEs, promote progression, and are associated with unfavorable outcomes in many cancers. Weaver et al. (2022) [42] reported that in murine models transplanted with colon cancer, CRC, bladder, and PDAC cell lines, inhibiting CCR8, a chemokine receptor expressed on tumor-infiltrating T_regs,_ using complementary monoclonal antibodies resulted in T_reg_ depletion and consequently an increase in CD8^+^ T-cell infiltration, as well as significantly improved anti-PD1 efficacy in combination. Collectively, CCR8 inhibition showed significant tumor regression [42,43], and because CCR8 blockade induces an immunological shift toward antitumorigenic responses, it may represent a promising therapeutic strategy in cancer treatment (Figure 1).

The presence of intratumoral T_regs_ expressing the Forkhead Box Protein (FOXP3) transcription factor (FOXP3^+^ T_regs_) in PDAC is associated with tumor progression, immune evasion, poor prognosis, and reduced intratumoral CD8^+^ T-cell abundance [44,45]. High FOXP3^+^ T_reg_ accumulation in regions surrounding the tumor may suppress local antitumor immunity by limiting CD8^+^ T-cell recruitment and function [45,46,47,48]. Consistently, pre-clinical targeting of FOXP3^+^ T_regs_ using anti-cytotoxic T Lymphocyte-associated-protein 4 (anti-CTLA-4), anti-CD25, and CCR5 inhibition reduced tumor growth and enhanced CD8^+^ T-cell-mediated antitumor responses [48]. Furthermore, Kiryu et al. (2021) [49] showed that patients with an immune rich TME possessing a high density of tumor infiltrating lymphocytes (TILs; D3^+^, CD4^+^ and CD8^+^ T-cells) in the TME that were positive for PD-1 in surgically resected PDAC tissues had significantly better overall survival (OS: 1086 days with high CD3^+^ T-cells; 1360 days with high CD4^+^ T-cells; 1223 days with high CD8^+^ T-cells) and disease-free survival (DFS: 554 days; 554 days; 607 days) compared to those with PD-1^-^ T-cells (OS: 699 days, DFS median: 351 days with high CD3^+^ T-cells). However, immune-rich TMEs in the presence of FOXP3 showed opposing results; patient resections with high levels of intratumoral FOXP3^+^ T-cells in the presence of PD-1^+^ T-cells showed significantly worse prognosis (OS = 692 days) as opposed to subgroups with low FOXP3 (OS = 1420 days) [49]. Collectively, these findings highlight that the prognostic benefit of T-cell-rich PDAC microenvironments is highly context-dependent, as FOXP3^+^ T_regs_ not only interfere with effective CD8^+^ T-cell infiltration but may also functionally suppress infiltrating cytotoxic T-cells once present within the tumor (Figure 1). Consequently, even “hot” PDAC tumors may remain therapeutically resistant in the presence of high FOXP3^+^ T_reg_ abundance.

Independent of immune cells, *FOXP3* gene expression has also been reported within PDAC [50]. Tumor cell-intrinsic FOXP3 can modulate cytokine expression (including effects on IL-6 and IL-8) and, in co-culture with naïve T-cells, FOXP3^+^ PDAC cells inhibit T-cell proliferation. These effects are partially reversed when cancer-cell FOXP3 is suppressed, supporting a role for cancer-derived FOXP3 in tumor immune evasion [44]. Wang et al. (2017) [51] further discovered that FOXP3^+^ PDAC cells directly trans-activate the T-cell chemotactic protein CCL5, promoting the recruitment of T_regs_ and contributing to immune evasion. Additionally, tumor-associated neutrophils secrete abundant CCL5, which enhances cancer cell migration and invasion while negatively correlating with cytotoxic CD8^+^ T-cell infiltration, further suppressing anti-tumor immunity [52].

Therefore, strategies aimed at enhancing CTL infiltration while simultaneously depleting or inhibiting FOXP3^+^ T_regs_ are likely critical for improving antitumor immune responses in PDAC. These strategies are best served by the use of 3D models that physiologically mimic both the 3D structure and physiological function of PDAC. For instance, a microfluidic PDAC system was used to observe variations in activated CD4^+^ and CD8^+^ T-cell infiltration towards tumor stimuli in different culture combinations. The microfluidic device was composed of PANC-1, PSCs and endothelial cells in adjacent chambers, which structurally and functionally mimics the in vivo PDAC TME, to which peripheral blood mononuclear cells (PBMCs) isolated from donor blood were added. By experimenting with different combinations of chambers, it was found that endothelial cells in the multi-culture system inhibited T-cell infiltration regardless of activation, a consideration critical to prevent overestimation and failure in clinical trials [53]. Similarly, the InterOMax model detailed in Section 4 also presented increased *FOXP3* expression and decreased T-cell activation when in the presence of human PSCs, as seen in in vivo PDAC tumors [54].

The density of the stroma further inhibits the host’s immune response to the tumor. It immobilizes infiltrating immunogenic cells such as monocytes, neutrophils and mast cells, as well as a small number of TILs, contributing to the “cold” phenotype of PDAC tumors [8]. It also leads to the collapse of the pancreatic vasculature, exacerbating hypoxia and free radical accumulation known to cause DNA damage and cell death, which in turn promotes cancer cell aggressiveness and contributes to the establishment of an immunosuppressive microenvironment [8,55]. Such conditions oppose the survival of immune cells and limit the infiltration of CD8^+^ cytotoxic T-cells, which are essential for antitumor immunity [16]. Hypoxia, alongside molecules produced by pancreatic cells like the cytokines CCL2 and GM-CSF, recruits an immunosuppressive environment containing T_regs_, and myeloid cells and differentiates them into their pro-tumorigenic phenotypes [8,15].

Overall, PDAC possesses multiple interconnected mechanisms that collectively maintain a highly immunosuppressive and poorly infiltrated TME. CAFs establish a dense desmoplastic stromal network that physically protects tumor cells while promoting immunosuppressive macrophage polarization, whereas FOXP3^+^ T_regs_ suppress effective intratumoral immune infiltration and anti-tumor responses (Figure 1). Compounding this, genetically distinct and heterogeneous sub-TME regions can generate diverse phenotypes, varying chemotherapy susceptibility and immune profiles within the same tumor. Additional factors, including hypoxia and inflammatory cytokines, further reinforce the immunosuppressive landscape. Consequently, PDAC remains exceptionally difficult to treat, with these features contributing to high mortality rates, poor prognosis and resistance to an array of therapies. Beyond host-derived influences, emerging evidence suggests that microbial components further modulate PDAC progression and therapeutic response, making PDAC more challenging to treat and emphasizing the urgent need for more clinically translatable and physiologically representative disease models.

## 3. The Microbiome’s Influence on Cancer

### 3.1. Microbial Signatures Correlate with Survival Duration and Treatment Response

Emerging evidence indicates that the patient’s gut, oral, and tumor-associated microbiome signatures are associated with oncogenesis, metastasis, and immune suppression in PDAC [56]. As with any association between bulk data and outcomes, interpretations of causality cannot be drawn without studying direct interactions, many of which can only be studied in models such as 3D organoid systems. Four questions must be answered to establish a connection between PDAC outcomes and the microbes identified from samples. First, is the microbe really present? Tumor microbiome studies have historically been less reliable in reporting colonization data than gut microbiome studies, with artifacts appearing in both the physical samples [57] and data interpretation [58]. Sample artifacts, typically due to bias introduced because the tumor microbiome has a very low microbial biomass, can lead to false positives [59]. Second, is the microbe truly colonizing the tumor? The identification of genetic material is an indication only of its presence; it is not the same as confirmation of colonization. If the microbe is present and alive, the third question becomes, what is it? Historically, 16S sequencing data reports at the genus level (or higher), which cannot explain species- or strain-level activity. There is a growing body of metagenomic work, which provides functional potential of the microbiome, but these remain bulk measurements that cannot fully match species and function. Finally, is the cancer microbiome a cause of the tumor, or is the tumor creating a milieu conducive to the growth of those microbes? Identifying the directionality of the association is critical to forming hypotheses about the role of microbes in PDAC and their potential use for treatment.

Beyond these issues of association, there is an additional upstream confounding factor: how representative of the community structure is a given sample? For gut microbiome assessment, stool samples are used. However, these are only representative of the rectum and do not exactly match what is found in the small or large intestines [60]. Furthermore, there is significant variation in stool composition, even within the same person on consecutive days [61], that can further confound associative interpretations. Importantly, exogenous factors also drastically influence the structure of the microbiome found in stool samples. These include diet, physical activity, body mass index, medication, disease states, and host genetic variations [62]. When considering a population like PDAC patients who have potentially been treated with chemotherapy and/or antibiotics following surgery, it is critical to consider the possibility that the tumor microbiome could be significantly impacted by these confounding factors, making models that incorporate the MTI axis even more necessary.

Deviation from healthy microbial gut and oral microbiome populations, known as dysbiosis, are frequently observed in PDAC patients [63]. Several studies have identified microbial patterns in oral and fecal samples unique to PDAC patients using shotgun and 16S rRNA sequencing (Supplemental Table S1). These studies reported that PDAC patients—in comparison to healthy controls—had enriched bacterial families such as Veillonellaceae [63,64], Bacteroidaceae, Lachnospiraceae G7, Enterobacteriaceae, and Staphylococcaceae [65], and genera such as *Akkermansia*, *Odoribacter* [64], and *Streptococcus* [63], with reductions in the bacterial families Ruminococcaceae, Lachnospiraceae, the bacterial order Clostridiales [64], the genus *Haemophilus* [65], and species like *Faecalibacterium prausnitzii* [63], *Streptococcus mitis* and *Neisseria elongata* [66] in oral and fecal samples. In addition to changes to the commensal bacteria, pathogenic bacterial populations have been implicated in tumor initiation. Two studies linked periodontal disease-causing bacteria, such as *Porphyromonas gingivalis* and *Aggregatibacter actinomycetemcomitans*, to a higher risk of PDAC development [67,68]. While there is consensus across these studies that bacterial in the gut and oral microbiomes are associated with PDAC, there is a lack of understanding around the mechanism. Whether or not these associations are causal, and if so, how specific species enact their effect, requires further study.

**Figure 2:**
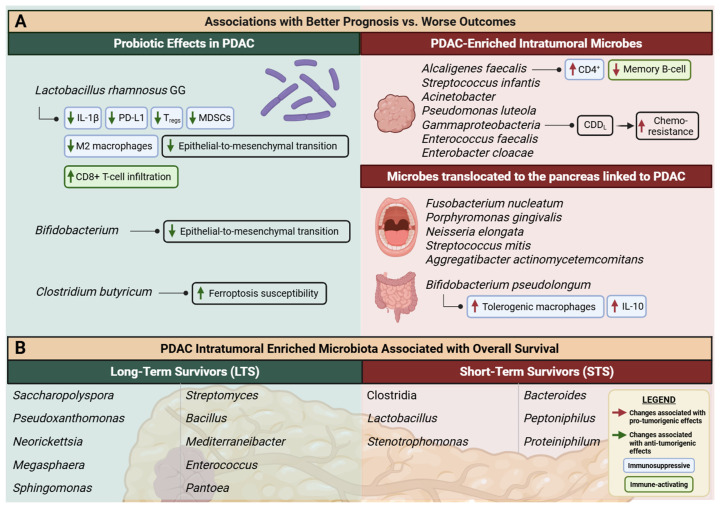
Influences of the intratumoral and translocated microbiome on PDAC. (**A**) Bacteria associated with anti-tumorigenic effects, long-term survival, and improved ICI efficacy (green panel, left-hand side). Bacteria associated with pro-tumorigenic effects, short-term survival, and worse ICI efficacy (red panel, right-hand side). (**B**) PDAC intratumoral microbes comparably enriched in LTS and STS. Up arrows indicate increased expression, down arrows indicate decreased expression. Abbreviations at the end of the article.

In addition to the systemic influences of the oral and gut microbiomes, these communities impact PDAC directly through the pancreatic microbiome. The pancreatic microbiome is thought to be established by bacteria from the oral cavity [65] and gut [69], which translocate to the pancreas via the circulatory system or the biliary and pancreatic ducts (Figure 2). Translocation of these species into the pancreas may then promote inflammation and cause acute pancreatitis development [70,71], which can increase the risk of PDAC [67]. It was further suggested that the progressive inflammatory nature of the PDAC TME may also promote this translocation and facilitate microbial colonization within pancreatic tissue [72]. Compared to healthy participants, the microbiome of PDAC has reduced microbial diversity [70], fewer butyrate-producing taxa, and a concomitant increase in pathogenic lipopolysaccharide (LPS)-producing bacteria [73], likely a reflection of the dysbiotic state of the gut- and oral-microbiomes of the patient (Supplemental Table S1). Importantly, no microbial differences between malignant and benign human surgical resections have been identified [74], implying that the intratumoral microbiome’s composition is established before malignancy.

The microbial composition of PDAC tumors has been linked specifically to survival duration and therapeutic response, though these associations are clouded by the disruptive effects of chemotherapy and radiotherapy on the microbiome [11,75,76]. Specifically, intratumoral microbial composition correlates with PDAC survival (Figure 2): long-term survivors (LTS, >5 years) of PDAC harbor more diverse and compositionally distinct microbiomes compared to short-term survivors (STS) [77]. For LTS, these genera include *Saccharopolyspora*, *Streptomyces*, *Pseudoxanthomonas*, *Bacillus clausii* [77], *Neorickettsia* and *Mediterraneibacter* [78]. Further studies in a Chinese cohort also identified intratumoral *Megasphaera*, *Enterococcus* and *Sphingomonas* positively correlated to increased overall survival. Huang et al. (2022) [79] found that *Megasphaera* sp. XA511 isolated from PDAC tumors was associated with LTS and improved antitumor efficacy of anti-PD1 therapy in a murine 4T1 breast cancer model. STS tumors, on the other hand, were dominated by Clostridia [77], *Bacteroides*, *Lactobacillus*, *Peptoniphilus* [80], and *Stenotrophomonas* [78]. Additionally, other taxa such as *Streptococcus infantis*, *Acinetobacter* spp. (*A. johnsonii*, *A. lwoffii*), and *Pseudomonas luteola* have been associated with poor patient outcomes (Figure 2), further highlighting microbial associations with outcomes [81]. Collectively, these insights point to tumor-associated microbiota as potential modulators of therapy response and/or prognostic markers (Supplemental Table S1).

### 3.2. Microbiome Influences on Immunity in PDAC

Building on evidence that sequencing studies have consistently associated microbial composition with survival duration in PDAC patients, immune–microbe interactions may help explain the observed correlations with patient prognosis, therapeutic responses and overall disease outcomes (Figure 2). Research across different cancer types has shown that the level of CD8^+^ T-cells within a tumor serves as a key indicator for predicting the effectiveness of anti-PD-1/PD-L1 immunotherapy [16]. Riquelme et al. (2019) [77] reported that LTS-associated bacteria from surgically resected PDAC samples, including *Saccharopolyspora*, *Pseudoxanthomonas*, and *Streptomyces*, correlated positively with intratumoral CD8^+^ T-cell and Granzyme B densities, suggesting that these microbes may enhance antitumor immune responses by fostering T-cell activation and infiltration. In contrast, the presence of STS-related anaerobic genera in PDAC tumor samples, such as *Bacteroides*, *Lactobacillus*, and *Peptoniphilus*, correlated with reduced tumor-infiltrating CD4^+^, CD8^+^, and CD45RO^+^ T-cells and shorter overall patient survival [80].

Intratumoral bacteria have been associated with immune responses within the TME, as briefly discussed above. In metastatic renal cell carcinoma, ICI treatment responders and non-responders showed notable differences in the relative abundance of intratumoral microbes [82]. In another study, Gopalakrishnan et al. (2018) [83] demonstrated that increased diversity in the gut microbiota can improve immunotherapy responses in metastatic melanoma by promoting T-helper cell-1 (Th1) polarization of CD4^+^ T-cells and enhancing the infiltration of cytotoxic CD8^+^ T-cells. 

Importantly, these studies are outside of the PDAC context and therefore cannot be directly translated. While the PDAC microbiome-tumor-immunity remains underexplored, there are some studies connecting our understanding of other cancers to PDAC. In an in vitro system, PDAC spheroid models infected with the oral bacterium *F. nucleatum* mechanistically linked to CRC initiation, progression, and treatment response [84], and induced the secretion of pro-tumorigenic cytokines such as GM-CSF, CXCL1, IL-8, and MIP-3α, which significantly enhanced PDAC cell proliferation and migration, with no observable effect in non-cancerous pancreatic epithelial cells. These tumorigenic effects were largely abrogated by GM-CSF blockade [85]. Taken together, the gut-, oral- and tumor-associated microbiota of PDAC patients likely affect the immune response (Figure 2). Though more research is needed to understand these processes, some mechanisms are understood.

#### Bacterial Metabolites

Accumulating evidence suggests that microbial-derived metabolites may be key mediators of the immunomodulatory effects of microbiota in PDAC. The intratumoral pancreatic microbiome can influence the TME and PDAC prognosis through these metabolic products, as well as other yet undefined mechanisms [79]. Microbial metabolites are small molecules produced by bacteria through primary or secondary metabolism, including bacterial cell-intrinsic components such as LPS or cell-extrinsic products, like short-chain fatty acids (SCFAs). These bioactive compounds can reshape the TME by modulating innate immune signaling, myeloid cell function, and T-cell infiltration, thereby impacting tumor progression, therapy resistance, and response to immunotherapy. Some microbes may also reduce treatment effectiveness by impairing immune responses or metabolizing therapeutic agents. Understanding these molecular mechanisms is critical for improving current cancer treatment strategies [86,87].

Specific species and their metabolites have been mechanistically tied to outcomes in other cancer types, though this area of research is still in its infancy. In CRC, the Gram-negative gut bacterium *Muribaculum gordoncarteri* and elevated levels of its metabolite urocanic acid suppressed MDSC recruitment through inhibition of the CXCL1–CXCR2 signaling axis in colorectal tumor vascular endothelial cells. Notably, ICI responders displayed higher fecal urocanic acid concentrations and increased abundance of *M. gordoncarteri* compared with non-responders [88], underscoring the translational potential of microbiome–drug interactions in modulating antitumor immunity and highlighting both as potential predictive biomarkers for treatment response. In addition to the role of urocanic acid, LPS likely plays a role in this species’ effectiveness.

LPS, an outer membrane component of Gram-negative bacteria, exerts bidirectional effects on tumor immunity, playing a pivotal role in pancreatic tumorigenesis. In PDAC-associated dysbiosis, the relative abundance of LPS-producing genera such as *Prevotella*, *Hallella*, and *Enterobacter* is elevated, compared to healthy controls [73]. Toll-like receptor (TLR)-signaling plays a major role in regulating inflammatory responses and is critically involved in tumorigenesis, immune suppression and therapy resistance [89]. LPS binds to and activates TLR4 on the tumor cell surface to stimulate tumor cell proliferation through a MyD88-dependent mechanism [90]. In support, Ikebe et al. (2009) [91] demonstrated that LPS enhances invasive capacity in pancreatic cancer cell lines via activation of the TLR4/MyD88/NF-κB inflammatory signaling pathway, with blockade of any element in this pathway effectively reducing LPS-induced tumor invasiveness. This suggests that the inflammatory TME in PDAC is influenced by the microbiome and sustained through LPS-induced TLR signaling. In melanoma, clinical responders to anti-PD-1 therapy were enriched for LPS-producing bacteria encoding the immunostimulatory hexa-acylated LPS, which elicited stronger TLR4-mediated immune activation and enhanced anti-PD-1 treatment efficacy in murine models, whereas penta-acylated LPS inhibits this activation [92]. Although LPS is capable of increasing TILs in murine PDAC xenograft models and pathways involving TLRs are predominantly pro-inflammatory, a positive correlation between TLR4 expression and PD-L1 expression was also reported [93,94]. Chronic stimulation through PD-L1 leads to T-cell exhaustion and indicates that chronic LPS–TLR4 activation promotes immune checkpoint-mediated immunosuppression in addition to its pro-inflammatory effects [93,94].

These contrasting immunological outcomes may reflect the time-dependent effects of LPS on the TME. Short-term LPS exposure promotes pro-inflammatory M1 macrophage polarization, whereas prolonged stimulation (>72 h) expands immunosuppressive M2-like macrophages and MDSCs, limiting CD8^+^ T-cell infiltration and reinforcing the immunosuppressive TME [93]. Interestingly, depletion of LPS-producing bacteria with polymyxin B significantly enhanced anti-PD-L1 efficacy in murine PDAC models despite evidence that LPS–TLR4 signaling can increase PD-L1 expression [93]. This suggests that the net effect of chronic LPS exposure is immunosuppressive if pro-inflammatory, as LPS simultaneously promotes M2 macrophage polarization, MDSC accumulation, and reduced cytotoxic T-cell infiltration, thereby limiting the effectiveness of ICI. Consequently, reducing intratumoral LPS may improve anti-PD-L1 responses by restoring a more immune-permissive microenvironment rather than simply altering PD-L1 expression levels. Collectively, these findings demonstrate that the effects of LPS on PDAC are highly context-dependent, influenced by microbial composition, LPS structure, and duration of exposure. 

Importantly, TLR4 activation is not restricted to LPS alone. For instance, Gram-positive *Bifidobacterium pseudolongum* has been shown to accelerate PDAC oncogenesis in a TLR4-dependent manner, as blockade of downstream signaling (e.g., via TRAF6 inhibition) abrogates its tumor-promoting effects. Cell-free extracts from *B. pseudolongum* promote macrophage polarization toward a tolerogenic phenotype characterized by increased anti-inflammatory IL-10 production, further supporting a role for microbiome-driven TLR signaling in shaping an immunosuppressive TME [69].

Additionally, certain *Lachnospiraceae* produce MHC class I peptides via flagellin-related genes, which are structurally homologous to tumor antigens, primed CD8^+^ T-cells and improved antitumor immunity against melanoma in ICI complete responders [95]. Other microbial metabolites, such as *Bifidobacterium*-derived inosine [96] and *Enterococcus* muropeptides [97] have also been implicated in improved responses to ICI in CRC and melanoma murine models, respectively.

Collectively, these findings highlight microbial-derived metabolites as key mediators of immune modulation and inflammation that might be useful therapeutics within the PDAC TME. The presence and functional activity of specific microbial taxa, such as LPS-producing bacteria, may be predictive of patient response to therapy. As many of these effects are mediated through interactions between microbial products, immune cells, and the TME, they cannot be adequately recapitulated in conventional 2D monocultures. Incorporating microbial-derived LPS into heterotypic 3D PDAC models containing tumor, stromal, and immune components may therefore provide a more clinically relevant platform for investigating microbiota-driven immune regulation, therapy response, and resistance mechanisms. Further studies are needed to understand the directionality of these associations, with particular attention to understanding the potential negative side effects of using bacteria as therapeutic adjuvants for PDAC immunotherapy.

The presence of specific microbial taxa has divergent immunomodulatory implications, influencing both T- and B-cell infiltration through TCR- and BCR-mediated signaling. In PDAC, B-cell infiltration has been associated with opposing clinical outcomes, contributing to a favorable prognosis through antibody-mediated anti-tumor responses, while also promoting tumorigenesis and immune suppression when induced by IL-1β, resulting in reduced survival [81,98]. The microbial context-dependent effect is exemplified by the genus *Alcaligenes*. *Alcaligenes* spp. can stimulate immune responses by activating dendritic cells and naïve B-cells via the lipid A component of LPS within gut-associated lymphoid tissues. However, *Alcaligenes faecalis* in resected early-stage PDAC is associated with increased naïve CD4^+^ T-cells and reduced memory B-cells, an immune profile linked to poor overall survival [81].

By shaping adaptive immune pathways and producing metabolites or peptides that mimic tumor antigens or alter the TME, microbes can either promote or suppress antitumor immunity. Growing evidence indicates that the composition of a patient’s microbiome can serve not only as a predictor of disease but also as a therapeutic immune modulator to enhance the efficacy of PDAC immunotherapy. Integrating microbial signatures and employing microbial therapeutic strategies into clinical stratification may improve prognosis prediction and optimize therapeutic responses and survival of PDAC patients towards both immunotherapy and chemotherapy [72].

### 3.3. Microbial Therapeutic Strategies and Experimental Investigations

As the influence of the tumor and gut microbiomes on PDAC outcomes becomes more apparent, their therapeutic manipulation emerges as a new treatment avenue. There are three main types of microbial therapeutic approaches in PDAC, which are commonly used in combination with each other and other treatments: first, the depletion of pro-tumorigenic bacteria using antibiotics; second, the administration of probiotics, prebiotics or their bioactive metabolites known as postbiotics; and third, fecal microbiota transplantation (FMT) from long-term PDAC survivors or healthy donors. Table 1 shows a summary of experimental investigations carried out and their observations across these strategies.

#### 3.3.1. Pro-Tumorigenic Bacterial Depletion

In order to understand the directionality and establish causality of the bacteria associated with poorer clinical outcomes in PDAC patients, animal models with antibiotic depletion of microbes or gnotobiotic mice have been utilized. In experimental mouse models, varying degrees of synergy with ICIs and chemotherapy have been demonstrated. In the wild-type BxPC3 and mutant L3.6pl variant of the *Kras* oncogene cell-line xenografts and genetically engineered *Kras^G12D/+^*; *PTEN^lox/+^*; *Pdx1-Cre* (KPP) mouse models, antibiotic-mediated depletion of intestinal microbiota slowed PDAC tumor progression, showed a decrease in poorly differentiated tumors, and resulted in fewer malignant lobules relative to microbiota-intact mice. The xenograft tumors themselves, however, regardless of antibiotic treatment, remained devoid of detectable intratumoral bacteria and did not establish them over time. It was suggested that gut microbiota or other commensals external to the tumor, rather than tumor-resident species alone, may influence disease trajectory. Intratumoral bacteria may therefore have more impactful roles associated with therapeutic response as opposed to tumor progression [74].

More strikingly, ablation of the gut and intratumoral bacteria was shown to overcome treatment resistance to both gemcitabine [99] and ICI [69] in CRC and PDAC models, respectively. Furthermore, antibiotic-mediated depletion of the gut microbiome protected against both preinvasive and invasive PDAC, whereas FMT from PDAC-bearing murine hosts, but not controls, reversed tumor protection. Bacterial ablation reprogrammed the immune environment with reduced accumulation of MDSCs, enhanced pro-inflammatory activation of macrophages (M1 polarization), and increased activation of CD4^+^ T-helper cells and CD8^+^ T-cells [69]. The method may not be directly applicable to humans, as antibiotics have off-target effects that may increase adverse outcomes in patients already inundated with antimicrobials and chemotherapeutics. However, it does serve as a proof-of-concept for establishing a role for microbes in cancer immunotherapy efficacy.

#### 3.3.2. Pre-, Pro- and Postbiotics

The identification of microbial communities in responders to ICI treatment has been useful as predictors of therapy response across multiple cancer types [100], which has opened avenues towards using microbes as probiotics in tandem with therapies in patients with poor treatment response [101]. Probiotics are the administration of live microorganisms in controlled doses that provide health benefits to the host. One of the biggest obstacles to using probiotics in translational studies, beyond the lack of mechanistic data linking the presence of microbes with functional outcome, is that association studies typically present microbial taxa at the Order or Family level, while microbes can only be administered at the species level.

While further work is needed to identify species-level probiotics efficacious in PDAC, it is a promising area. Xie et al. (2025) [102] introduced *Clostridium butyricum*, known to be an intratumoral bacterium of the human gut and is commonly in reduced abundance in fecal samples of PDAC patients, as a probiotic in CRC murine models [103]. The introduction of *C. butyricum* in germ-free and immunologically humanized mouse models resulted in the inhibition of the PI3K-AKT-NF-κB-IL-6 cascade signaling, associated with tumor progression and inflammation. This occurs by the interaction between the *C. butyricum* surface protein secD and the CRC receptor GRP78. The resulting reduction in IL-6 in combination with anti-PD1 further improved CD8^+^ T-cell infiltration and suppressed TAM infiltration in the murine models, as well as heterotypic organoid models, inclusive of matched stromal CAFs and immune cells [102]. Similarly, oral gavage supplementation of *C. butyricum* or its metabolite butyrate in PDAC murine models increased tumor susceptibility to ferroptosis, a form of programmed cell death, through enhanced intracellular oxidative stress and accumulation of intracellular lipids [104]. 

*Clostridium sensu stricto 1* was associated with reduced risk of PDAC development [105]. However, this same species was also identified as highly abundant intratumorally in STS PDAC patients [79]. These differing observations, distinguished primarily by the location of the *Clostridium* spp., suggest that bacterial location and surrounding microenvironment may strongly influence microbial behavior in addition to any species- or strain-specific differences. Within the gut, Clostridiales have access to fermentable substrates required for beneficial butyrate production, whereas within the devascularized and immunosuppressive PDAC TME, the same bacteria may adopt context-dependent pathogenic or tumor-supportive roles. This further suggests that microbial abundance alone, particularly from FMT stool profiling, may not fully reflect microbial function within the intratumoral environment. A similar phenomenon has also been reported with *Enterococcus faecalis*, where its muropeptides enhanced ICI efficacy in melanoma models. [92]. However, when introduced to the hypoxic environments within PDAC spheroids, both *E. faecalis* and *Enterobacter cloacae* increased pathogenicity [14] (Figure 2A). Whether direct supplementation of postbiotics could counteract these tumor-promoting effects in STS-associated PDAC models remains an important area for investigation.

The administration of *Bifidobacterium*- and *Lactobacillus*-enriched probiotic mixtures has shown promising results (Figure 2A). In PDAC-xenografted mice, these mixtures attenuated epithelial-to-mesenchymal transition (EMT), a phenotypic shift that drives metastasis, thereby limiting tumor invasiveness [106,107]. Han et al. (2024) [93] also used the probiotic potential of *Lactobacillus rhamnosus* GG to improve immunotherapeutic efficacy in PDAC murine models, which is discussed further in Section 3.4. These studies suggest that supplementation with different probiotic species may exert distinct anti-tumorigenic effects.

Prebiotics are commonly defined as substrates digestible by host microbes that confer health benefits. Non-digestible carbohydrates, such as inulin, are fermented by gut microbes, producing SCFAs, like butyrate and propionate, that play many roles in gut health, immune modulation and cancer dynamics [108]. Prebiotics attenuated tumor growth and, in combination with inhibitor drug therapies, like MEK inhibitor therapy, reduced tumor resistance to treatments in melanoma and colon cancer [109]. The use of inulin as a prebiotic is sometimes used in combination with other pre- and probiotic regimens. Inulin with prebiotic mucin in melanoma and colon cancer [109], and probiotics such as *Lactobacillus plantarum* LS/07 in colon cancer [110] induced apoptosis, reduced intratumoral pro-inflammatory cytokines, and also increased dendritic cell, CD4^+^ and CD8^+^ T-cell infiltration in rat and murine models. Furthermore, the addition of inulin enriched the growth of microbial taxa with antitumor potential, such as *Bacteroides*, *Akkermansia muciniphila*, *Parabacteroides* and *Barnesiella*, as well as *Clostridium* spp. known to produce butyrate [109].

In a PDAC randomized clinical study, oral administration of a probiotic group consisting of 10 non-pathogenic strains within the genera *Streptococcus*, *Bifidobacterium*, and *Lactobacillus*, along with inulin prebiotics, collectively termed the synbiotic group, conferred anti-tumorigenic responses and significantly decreased adverse effects post-surgery compared to probiotic treatment alone or the placebo groups. Two weeks prior to and after the pancreaticoduodenectomy surgical procedure, two capsules of probiotic supplements were administered once a day, alongside two capsules of 1000 mg inulin. The symbiotic group had significant increases in CD8^+^ T-cell infiltration and anti-tumorigenic interferon-γ (IFN-γ) expression. The synbiotic and probiotic groups exhibited a gradual decrease in inflammatory cytokines compared to the placebo groups, associated with reduced postoperative complications [111]. Considering the above findings, oral supplementation with prebiotics is a simple, non-invasive strategy to improve the tumor immune microenvironment in combination with similar treatments like probiotics or conventional therapies and has already been supported with clinical application. Potential benefits observed with inulin may also extend to other prebiotic compounds and therefore warrant similarly rigorous investigation.

In addition to pre- and probiotic administration, emerging interest surrounds the use of postbiotics as supplemental treatments to chemotherapy and immunotherapy [108]. Postbiotics are non-viable microbial components and secreted bioactive compounds resulting in prebiotic metabolites. Consequently, some of the therapeutic benefits associated with synbiotic or combined pre- and probiotic regimens may ultimately be mediated through postbiotic production. Among these are SCFAs, which are also associated with potent tumor-suppressive effects [106]. Donohoe et al. (2014) [112] demonstrated that butyrate, the bacterial SCFA, functions as a nuclear histone deacetylase (HDAC) inhibitor in CRC, promoting histone acetylation, apoptosis, and tumor suppression in gnotobiotic mouse models under pre- and probiotic interventions. Gnotobiotic BALB/c mice repopulated with 4 commensal bacteria from the altered Schaedler flora, and with or without butyrate-producing *Butyrivibrio fibrisolvens* (probiotic), were fed either low- or high-fiber diets (prebiotic). CRC was then induced with azoxymethane (AOM) and dextran sodium sulfate (DSS) after bacterial supplementation. Only when in combination with the high-fiber diet and the probiotic *B. fibrisolvens*, mice developed significantly fewer tumors compared to other treatment groups (1 vs. 3–4 tumors per mouse), with this protective effect remaining evident even under higher AOM/DSS doses (3 vs. 8–11 tumors per mouse) [112]. This data suggests that probiotics or prebiotics alone may not be sufficient to fully protect against tumorigenesis. However, this study model was limited to only four commensal bacterial species and therefore lacked the complexity of microbial crosstalk observed in the clinical gut environment.

Clinical translation of postbiotics remains constrained by limited mechanistic understanding and a lack of standardization in therapeutic applications [106]. Nevertheless, similar observations have also been reported in PDAC. For instance, sodium butyrate in combination with chemotherapy conferred similar anti-tumorigenic results [113]. In PDAC cells, the addition of butyrate in combination with gemcitabine inhibited cell growth and increased apoptosis. In subcutaneous PDAC murine xenografts, butyrate with or without gemcitabine significantly decreased polarization of M2 macrophages and desmoplastic stroma. Butyrate also influenced the increased abundance of SCFA-producing bacteria and reduced pro-inflammatory microbes in feces [113], as seen similarly in clinical PDAC patient fecal samples [114]. Using 16S rRNA gene sequencing on PDAC tumoral resections, PDAC samples showed decreased intratumoral abundance of the butyrate-producing genus *Jeotgalicoccus* [115] and the phylum Actinobacteria [116] compared to matched normal pancreatic tissues and healthy controls, respectively. These findings suggest that butyrate production may play an important role as a postbiotic in limiting tumorigenesis and improving therapy response. 

Beyond its anti-tumorigenic effects, butyrate also appears to contribute to broader SCFA homeostasis within the host by increasing other SCFA-producing bacteria in the gut, supporting overall microbial and physiological health. Butyrate-producing species such as *Faecalibacterium prausnitzii*, *Eubacterium rectale*, and *Roseburia intestinalis* found in the mucosal gut environment have emerged as promising probiotic candidates due to their roles in maintaining intestinal immune balance and exerting butyrate-mediated anti-tumor effects [106]. These beneficial taxa are significantly reduced in the gut environment of PDAC patients relative to healthy controls, suggesting a link between microbial variation and disease progression [63,114]. Restoration of such communities or their metabolic outputs in PDAC may therefore serve as an effective therapeutic strategy.

#### 3.3.3. Fecal Microbiota Transplantation (FMT)

The composition of gut microbial communities differs between healthy individuals and patients with PDAC and has been associated with variations in patient survival outcomes. As these microbial communities are transferable between individuals via FMT, a strategy that has shown therapeutic efficacy in gastrointestinal disorders, FMT has emerged as a potential approach for modulating the microbiome in PDAC. Riquelme et al. (2019) [77] found that in antibiotic-treated tumor-bearing mice (C57BL/6 implanted orthotopically with KPC cell lines), FMT from human LTS or healthy donors not only slowed tumor progression but also enhanced immune activation of CD8^+^ T-cells. The same FMT treatments also reduced tumor infiltration by T_reg_ cells, thereby shifting the immune balance towards an anti-tumor state. Mice that received FMT from STS patients had larger tumors compared to healthy FMT controls [77]. Furthermore, the FMT STS mouse group developed intratumoral microbial profiles enriched in Clostridiales, closely reflecting the donor tumor microbiota. Notably, Clostridiales was identified as the strongest microbial signature associated with PDAC STS patient tumors compared with LTS tumors, with the highest differential abundance [77] (Figure 2B).

While FMT-based murine models have provided valuable insight into microbiome–tumor interactions in PDAC, their ability to accurately recapitulate the human intratumoral microbiome remains limited. In a study transplanting human-derived microbial communities into antibiotic pretreated patient-derived xenograft (PDX) mice, the human-derived bacteria only partially colonized the murine gut (~40% transferred from human donor) while only a small fraction of gut microbes directly translocated to the tumor (<5% of human origin and 20% of murine origin), with the majority of intratumoral bacteria arising from the endogenous, murine microbiome [77]. Despite minimal translocation, FMT still significantly altered the composition and diversity of the tumor microbiome, indicating that microbiome-driven effects may occur predominantly through indirect modulation rather than direct bacterial seeding into the tumor. This distinction suggests that FMT in murine models reflects a reshaping of the tumor-associated microbial ecosystem rather than faithful reconstruction of the human condition. Consequently, while these models are widely used to investigate microbiome-mediated immune and tumor responses, species-specific differences and incomplete representation of the clinical intratumoral microbiome may limit their translational relevance to PDAC progression and therapy response. This highlights a critical need for more representative and clinically translatable models that better capture the complexity and origin of the human intratumoral microbiome.

Even though human-derived fecal microbiota has shown only partial transferability and immune reconstitution in murine models, transgenic murine systems have more recently been experimentally utilized to investigate microbiome-driven PDAC progression through FMT. In one study, fecal microbiota from inflammation-induced, advanced-stage double-transgenic *Kras/Ela-CreERT* (Kras/Cre or KC) PDAC mice were transplanted into unstimulated recipient mice harboring the same Kras/Cre mutation. Recipient mice subsequently developed macroscopic tumors, exhibited progressive reductions in microbial diversity over time, and demonstrated distinct alterations in both stool and intratumoral microbial composition. These changes included increased abundance of *Faecalibaculum*, *Bifidobacterium*, mimicking microbiomes in advanced PDAC, alongside reductions in *Lachnospiraceae*, *Roseburia*, and uncultured *Desulfovibrionaceae*. *Lachnospiraceae*, *Roseburia*, and other *Firmicutes* taxa, which were reduced in advanced PDAC mice, are recognized producers of anti-inflammatory SCFAs, like butyrate. Reduced abundance of these microbial populations is consistent with microbial signatures previously associated with chronic pancreatitis and PDAC [117], suggesting the possible loss of anti-inflammatory microbial populations during disease progression. Together, these findings support a contributory role for gut and intratumoral microbiota in driving PDAC development within genetically susceptible hosts.

Although FMT has demonstrated promising therapeutic effects in human clinical trials and preclinical models of cancer, there remain critical obstacles to its use in cancer therapy. First and foremost, there is the issue of safety: while every effort is made to screen donors for pathogens, there is always the risk of transmitting an infectious agent with FMT [118], and cancer patients are already at a higher risk of infection due to their immunocompromised state. Additionally, there is an increased risk of bacteremia due to neutropenic status [119]. A potential unwanted side effect of the increased immune activation to aid immunotherapy is the onset or worsening of graft-vs-host disease or autoimmunity, seen in some studies using FMT to treat chemotherapy-induced colitis [120,121]. Finally, FMT’s goal is to repopulate the gut with a diverse microbiome, but this can lead to the introduction of pro-tumorigenic microbes or metabolites [122].

In all FMT work, selection of “healthy” donors, FMT delivery method, and engraftment remain ongoing issues in the field. Healthy controls are the standard donor source, but defining “healthy” remains debatable, and outcomes are variable between individuals, with risks of rejection and low engraftment. Alternatives include autologous FMT, where patients’ own stools are banked prior to disease onset, or using stool samples from patients who previously responded well to therapy, an approach that is gaining traction as an adjuvant in immunotherapy. Equally critical is the delivery route, where traditional methods such as enema, colonoscopy, and endoscopy are increasingly complemented or replaced by oral capsules [123,124,125,126], with evidence that combining routes can improve microbiome engraftment rates [127]. Even when the donor and the route of administration are matched to the recipient, FMT may not be successful, with engraftment of transplanted microbes low or undetectable [128]. Yet, despite these obstacles, FMT remains a promising avenue for treatment options in severe, unresponsive conditions. Experimentally, however, species-specific differences in gut microbiota composition between human and murine donors may hinder the faithful translation of microbiome-mediated immune responses and tumorigenic effects from murine models to humans [129]. Future studies must integrate the microbiome-tumor-immune axis to understand the mechanistic basis of FMT success so that personalized, rationally designed consortia rather than full microbiota transfer can be used.

### 3.4. Combination Therapies with ICI

Expanding on current understanding of the impact of bacteria and their metabolites on cancer immunotherapy, the field is beginning to explore using microbiota manipulation to improve ICI therapy in preclinical and clinical models. In the CRC model CT26, FMT enhanced the efficacy of anti-PD-1 therapy in tumor-bearing mice, prolonging survival and improving tumor control relative to FMT or anti-PD1 treatments on their own. *Bacteroides* species, including *B. thetaiotaomicron* and *B. fragilis*, have been implicated as key contributors to enhanced anti-PD1 efficacy [130]. Mechanisms include the induced secretion of anti-inflammatory IL-10 in dendritic cells, intestinal homeostasis and inhibited CRC growth through its SCFA propionate. In the same study, FMT downregulated tumorigenic metabolites and pathways, while upregulating metabolites with anti-tumor effects and inhibiting tumor-promoting microbes. FMT in combination with anti-PD1 resulted in a higher proportion of altered plasma metabolite production compared to monotherapies (anti-PD-1 or FMT), and upregulated the metabolite kynurenic acid, known to inhibit colon and renal cancer proliferation [130].

Probiotic-based approaches have also emerged as a therapeutic avenue for improved therapeutic outcomes (Figure 2A). To address PDAC resistance to immunotherapy associated with the modulation of intratumoral bacterial communities, Han et al. (2024) [93] orally administered the tumor-targeting bacteria *Lactobacillus rhamnosus* GG with a gallium (Ga^3+^)-polyphenol network as probiotics in murine models of PDAC. Gallium disrupts bacterial iron metabolism and iron-associated survival mechanisms. The *L. rhamnosus* and Ga^3+^ behave as biological antagonists towards intratumoral Gram-negative and LPS-producing bacteria. This interaction consequently depleted the pro-tumorigenic *Proteobacteria* and LPS produced by bacteria, inhibiting tumoral TLR4/NF-κB signaling pathway activation. Pathway inactivation resulted in reduced tumor cell expression of immunosuppressive IL-1β and PD-L1 receptors and upregulated expression of anti-tumor cytokines like IFN-γ, TNF-α, IL-6, and IL-12. These shifts improved CD8^+^ T-cell infiltration and significantly decreased intratumoral T_regs_, MDSCs and M2-like macrophages. Collectively, the probiotic disrupted the growth of the existing tumor, inhibited metastasis of PDAC tumors to the lungs, reduced the LPS-producing intratumoral microbiome, and established an antitumoral immune microenvironment [93]. A major limitation of this study is that the enhanced efficacy of anti-PD-L1 therapy following depletion of LPS-producing bacteria was demonstrated using the antimicrobial peptide polymyxin B rather than the proposed probiotic itself. Although the probiotic was shown to reduce PD-L1 expression, its effects were not directly evaluated in combination with anti-PD-L1 therapy.

Expanding beyond single-strain probiotic interventions, Zhou et al. (2026) [131] isolated and identified 15 bacterial species from the feces of non-small cell lung cancer (NSCLC) patients that responded to the ICI anti-PD1 therapy. The 15-species community was orally administered daily to specific-pathogen-free (SPF) female mice that were subcutaneously injected with melanoma, CRC, NSCLC, or primary squamous cell carcinoma cell lines. The multi-strain probiotic increased the efficacy of anti-PD1 treatment in all tumor models through increased tumor infiltration and cytotoxicity of CD8^+^ T-cells, as opposed to anti-PD1 therapy alone. Using a similar experimental setup, NSCLC model mice were pre-treated with antibiotics and orally supplemented with FMT from non-responder patients with advanced NSCLC and exhibited resistance to anti-PD-1 therapy as anticipated. However, supplementing the 15 beneficial bacteria by oral gavage into the non-responder FMT murine group in combination with anti-PD1 reversed therapy resistance and significantly reduced tumor growth [131]. These results suggest that the identification and implementation of selective beneficial bacteria, either individually or as communities, may be used as probiotics to reshape the TME from an immunologically “cold” to “hot” phenotype through complex synergistic mechanisms. In doing so, probiotics have the potential to serve as minimally invasive adjuvants that enhance the efficacy of existing immunotherapies in clinical settings.

Beyond commensals, engineering or modifying the intratumor microbiome to directly kill or deliver antitumor molecules is being considered as a therapeutic tool, though studies remain limited (Table 1). This is largely due to key gaps in understanding of the PDAC intratumoral microbiome, particularly in separating it from confounding contributions of oral and gut microbial translocation and distal manipulation. In addition, the highly desmoplastic PDAC TME and its effects on intratumoral microbial interactions and function remain unclear, with microbes likely behaving very differently under such extreme conditions. Leveraging the genetic and molecular interactions between tumor cells and microbes may allow selective targeting of pathogenic species or augmentation of beneficial populations [11], offering a promising microbiome-based approach for PDAC therapy.

**Table 1:** Summary of microbial therapeutic interventions conducted on human tissue samples, 3D and murine models in various cancer types.

Tumor	Model Type	Bacterial Intervention	Results	Reference
PDAC	*KrasG12D*;*Trp53^R172H^*; *Pdx1-Cre* (KPC) implanted C57BL/6 mice	FMT	Antitumor response and immune activation were enhanced with the FMT from LTS with no evidence of disease. FMT from LTS and healthy controls further decreased immunosuppressive T_reg_ tumor infiltration.	[77]
PDAC	Double-transgenic *Kras/Ela-CreERT* (KC) mice stimulated with Cerulein	FMT	FMT from KC mice with stimulated advanced PDAC to unstimulated KC mice changed stool and intratumoral microbial diversity over time and developed macroscopic tumors.	[117]
PDAC	Xenograft and the *KrasG12D*;*PTEN^lox^*; *Pdx1-Cre* (KPP) genetic mouse	Antibiotic treated	Microbial ablation slowed tumor progression, resulting in fewer malignant lobules than in microbiota-intact mice. Although xenograft cohorts had no intratumoral bacteria, PDAC tumor growth was sustained, suggesting that intrapancreatic microbiota is not the sole driver of PDAC acceleration.	[74]
PDAC	Human fecal and tissue samples and C57BL/6 (H-2Kb) mice (KC, KPC, OT-I, OT-II, WT)	Antibiotic depletion, overcoming gemcitabine and ICI resistance in mice	Bacterial depletion enhanced ICI efficacy by upregulating PD-1 expression, leading to reduced MDSCs and increased M1 macrophage differentiation, resulting in enhanced Th1 differentiation of CD4^+^ T-cells and CD8^+^ T-cell activation.	[69]
CRC	Gnotobiotic mice, AOM/DSS tumor initiation	Pre- and probiotic effects	Butyrate collects in the nucleus as a tumor-suppressive metabolite functioning as an HDAC inhibitor, stimulating histone acetylation, inhibiting proliferation and inducing apoptosis.	[112]
CRC	CRC organoid/CAF co-culture, germ-free mice, humanized mice	Probiotic	Enhance anti-PD1 efficacy by blocking IL-6 secretion, improving CTL infiltration and suppressing TAMs.	[102]
Breast	4T1 murine organoids with matched immune cell co-culture (iTO)	Bacterial metabolites & ICI (anti-CTLA-4 and anti-PD1)	Significantly enhanced ICI-led tumor apoptosis and increased CD8 mRNA and IFNγ expression on T-cells.	[132]
CRC	CT26 inoculated mice	FMT & ICI	Combination therapy resulted in improved tumor control and survival rate compared to single treatment. Tumor-bearing mice treated with ICI (anti-PD1) changed microbiota composition through FMT, wherein, from the *Bacteroides* genus, increased levels of *B. fragilis* and *B. thetaiotaomicron*, along with decreased levels of *B. ovatus*, may have enhanced anti-PD-1 efficacy. Upregulated bacterial metabolites post-FMT in mouse plasma are suggested to promote the anti-PD-1 response.	[130]
CRC	C57BL/6 mice	Antibiotic treatment	Hexa-acylated bacterial-derived LPS enhanced ICI anti-PD-1 efficacy with TLR4 signaling and increased tumor-infiltrating CD8^+^ T-cells. Antibiotic-induced depletion of hexa-acylated LPS bacteria inhibited anti-PD-1 tumor reduction. Penta-acylated LPS reduced immune activation and did not improve ICI therapy.	[92]
Colon	MC-26 cell line subcutaneous xenograft in immunocompromised BALB/c mice	Antibiotic treatment with a chemotherapy drug	Depletion of bacteria with the cytidine deaminase enzyme, like *Gammaproteobacteria*, using the antibiotic ciproflaxin and the chemotherapeutic drug gemcitabine, significantly reduced tumor size	[99]
PDAC	Orthotopic xenograft of C57BL/6 J mice using the KPC1199 cell line	Pro- and postbiotic	Mice were supplemented with *C. butyricum* supernatant or butyrate. Enhanced susceptibility to ferroptosis via intracellular oxidative stress and lipid accumulation, thus increasing anti-tumor potential.	[104]
PDAC	Subcutaneous xenograft of the BxPC-3 cell line into nude BALB/c mice	Postbiotics and chemotherapy drug	Butyrate with or without gemcitabine decreased desmoplastic fibrosis, significantly reduced tumor size, and protected the intestinal barrier. Shifted gut microbiome to have more butyrate-producing bacteria and fewer pro-inflammatory bacteria.	[113]
PDAC	Subcutaneous and orthotopic injection of PANC-02 cell lines into C57BL/6 mice	Probiotic and ICI	Supplementation with *Lactobacillus rhamnosus* GG embedded in a gallium-polyphenol network antagonistically competed with LPS, reducing TAMs and MDSCs, and increasing CD8^+^ T-cells, enhancing anti-PD-L1 efficacy.	[93]
Melanoma, Colon cancer	Subcutaneous injection of cell lines into gnotobiotic C57BL/6 and C3H mice	Prebiotic	Inulin and mucin both inhibited melanoma growth. Inulin, but not mucin, limited colon tumor growth and enhanced MEK inhibitor treatment efficacy. They both altered gut microbiota composition towards phylotypes with anti-tumor potential.	[109]
Breast cancer	Sprague-Dawley rats intraperitoneally injected with N-methyl-N-nitrosourea	Probiotic, Prebiotic, Melatonin	The probiotic *Lactobacillus plantarum* LS/07 and the prebiotic inulin, in combination with melatonin, enhanced CD4^+^ and CD8^+^ T-cell infiltration, but intratumoral CD25^+^FOXP3^+^ T_regs_ were also increased.	[110]
Melanoma, CRC, NSCLC, primary squamous cell carcinoma	Subcutaneous injection of cell lines into SPF female BALB/c and C57BL/6 mice	Probiotic community and ICI	15-bacteria consortium (RCom) increased tumor infiltration and cytotoxicity of CD8^+^ T-cells, compared to anti-PD1 therapy alone in all tumor types. NSCLC mice treated with FMT from non-responder ICI patients exhibited resistance to anti-PD-1 therapy, but RCom supplementation reversed therapy resistance and significantly reduced tumor growth.	[131]

Collectively, these findings position both gut and intratumoral microbiome modulation as a promising avenue to reshape tumor immunity, sensitize PDAC to existing therapies, and enhance treatment efficacy. While early results are encouraging, most of these approaches remain based on murine models, which do not fully reflect human PDAC biology. Thus, a key limitation remains the lack of robust, patient-relevant models that can simultaneously capture the intratumoral microbiome, heterogeneic desmoplastic stroma, and dynamic immune interactions under physiologically relevant conditions. Without such models, it is difficult to define causality, predict patient-specific responses, and identify reproducible microbial or metabolic targets. As a result, while microbiome-based therapeutic strategies in murine models show promise, their clinical translation remains constrained by variability across models and incomplete mechanistic resolution, highlighting the urgent need for clinically translatable preclinical models.

## 4. Translational Capability of Pre-Clinical Models

### 4.1. Two-Dimensional and Animal Models

**Figure 3:**
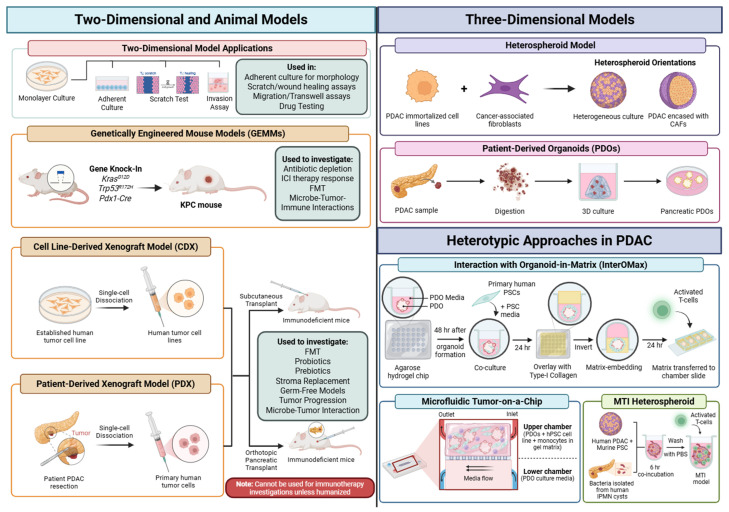
An overview of pre-clinical models used in PDAC research. Two-dimensional or monolayer cultures (red box) are commonly used for simplistic direct assays. Adherent monolayer cultures display morphological characteristics that may help improve understanding of specific cell types. Animal models include GEMMs and PDX murine models (orange boxes); GEMMs are formed from genetic knock-ins, whereas PDXs are established by injecting either commercially available immortalized human cell lines or dissociated cells from patient tumor resections. Injections are either done under the skin (subcutaneous) or into the pancreas (orthotopic). 3D models include multicellular aggregates called heterospheroids (purple box) or organoids formed from patient tissue samples (pink box). These models have been used in experimental systems involving multiple biological niches or heterotypic approaches; the InterOMax model and microfluidic tumor-on-a-chip both used PDOs, stromal cells and immune cells (light blue boxes). The MTI model (green box) used heterospheroids in combination with used cancer and immune cell lines in place of PDOs and incorporated bacteria into the model. Abbreviations at the end of the article.

Traditional 2D models involve culturing tumor-derived cell lines as monolayers on hard surfaces. These systems can also incorporate transwell-based assays, where cells are seeded into upper membrane chambers coated with ECM components such as collagen I (Figure 3). 2D models are cost-effective and highly manipulable, making them valuable experimental workhorses that have supported research for decades. However, when considering their physiological similarity to complex tumors like PDAC, 2D models lack key biological features limiting their utility to investigate PDAC cell motility, invasiveness, and responses to inhibitory compounds [133,134]. The most critically missing features are cell-type heterogeneity, oxygen gradients, ECM and stromal interactions, and tumor–microenvironment dynamics. These differences severely restrict 2D models’ predictive value for drug response and tumor biology studies [72,135,136].

In contrast, animal models provide a more physiologically relevant platform for studying pancreatic tumorigenesis and therapeutic responses than 2D models. Yet they have their own obstacles to clinical translation, as introduced above. Here, we will discuss the advantages and disadvantages of the two main types of animal models used in PDAC research, genetically engineered mouse models (GEMMs) and PDX models (Figure 3). GEMMs recapitulate key aspects of PDAC tumor initiation and progression, possess a desmoplastic stroma, and acquire the impaired vascularization that restricts drug delivery [137]. PDX models, on the other hand, maintain the structure of the patient tumor, including human-derived tumor and stromal cells, better recapitulating the relevance to drug efficacy [138,139,140,141]. Since PDX models lack an intact immune system, they cannot be used for the interrogation of tumor–immune interactions and immunotherapeutic strategies, while GEMMs can [142].

A commonly used GEMM of pancreatic cancer is the KPC model (Figure 3), which harbors pancreas-specific expression of *Kras^G12D^* and *Trp53^R172H^* mutations driven by the *Pdx-1-Cre* recombinase. KPC mice progress through all stages of PDAC and spontaneously develop invasive metastatic tumors, making them a widely used model of advanced disease [69,143]. Importantly, they recapitulate several hallmark features of human PDAC, including extensive desmoplasia, inter- and intratumoral TME heterogeneity, chromosomal instability, progressive adverse effects similar to cachexia, such as lack of appetite and muscle and fat loss, hemorrhage in the abdominal cavity, and metastatic spread [143]. As such, KPC models are regarded as the most clinically representative murine models of advanced PDAC currently available. Another GEMM model is the KC mouse that expresses mutant *Kras* alone under *Pdx-1*-*Cre* control without accompanying *Trp53* mutation, resulting in slower tumor progression and predominantly pre-invasive pancreatic intraepithelial neoplasm-like lesions [69,143].

Germ-free KC and KPC mice have also become valuable tools for investigating intratumoral microbial shifts and influences on tumor progression compared to microbial-intact KC or KPC mice [69]. KPC mice can also be cross-bred with transgenic mouse strains for a range of experimental applications, including depletion of specific stromal components such as α-SMA to investigate the role of CAFs within the PDAC microenvironment [23], or fluorescent labeling of Cre recombinase for tumor visualization studies [144]. Given that gut microbiota can translocate to the pancreas and influence the TME, FMT-based approaches have been used to assess gut microbial contributions to disease progression (Figure 3). Studies involving the transplantation of human donor feces into KPC mice [77], advanced-stage KPC-derived feces into pre-invasive KC mice [69], and inflamed KC microbiota in KC recipients [117] demonstrated measurable shifts in both gut and intratumoral microbial composition alongside altered tumor progression.

While GEMMs provide a histologically comparable representation of PDAC and can be modified and investigated using knockout and transgenic technologies [145,146,147], their utility is constrained by low experimental throughput, high costs, and the extended time required to establish and maintain these models [137,148]. Although FMT strategies allow manipulation of the gut microbiota in GEMMs, these models remain limited in their ability to faithfully reproduce the complex human intratumoral microbiome. As a result, investigations of intratumoral microbial contributions to PDAC are largely restricted to comparative analyses between germ-free and microbiota-intact murine systems rather than direct modeling of human intratumoral microbial composition. Therefore, these models lack utility for understanding interactions in the human-derived MTI axis.

PDX models theoretically address some of these limitations by directly implanting human PDAC tissue into immunocompromised mice, either orthotopically or subcutaneously (Figure 3). Subcutaneous PDX models are minimally invasive and easy to monitor, whereas orthotopic models more closely recapitulate primary human PDAC and metastatic progression, but are harder to assess during tumor progression [135,149]. While direct implantation of human tissue into PDX mice could potentially preserve characteristics of the donor tumor, including the intratumoral microbiome, this assumption remains unconfirmed, as matched human donor-to-PDX 16S rRNA microbial profiling studies are currently lacking. One study reported that intratumoral microbiota present in human PDAC tissue samples had significantly higher inter-sample divergence and microbial diversity as compared to PDX models [150]; however, the authors do not explicitly mention whether the intratumoral microbiome in PDX models was compared with that of their matched human donors. This possibility seems unlikely, however, as the dataset for each sample type was obtained from different sources.

PDX models retain the genetic, histologic, and differentiation features of patient tumors in situ and provide reliable predictive value for patient-specific drug responses [135,139]. Delitto et al. (2015) [140] reported that histological characteristics such as glandular formation and tumor differentiation in subcutaneous murine xenografts were retained from the primary PDAC patient, with persistent desmoplastic stromal components and metastatic circulating potential through several passages into new mice. Similarly, orthotopic mice shared and maintained the histological architecture, stromal fibrosis and mucin production of their original human donors across multiple murine passages [138,141]. However, the stroma was found to progressively lose human stromal components and be replaced by murine stroma after implantation [140].

While PDXs can model metastatic potential and accurate histological representations of their human PDAC counterparts, these models are more resource-intensive than GEMMs, suffer from variable engraftment success, and, quite importantly, are immunodeficient. Since PDX models rely on immunodeficient host mice that lack key functional immune components, their application in immunotherapy research is restricted [135,140] and therefore hinders the investigation of MTI interactions within this system. Certain studies have attempted to “humanize” murine models by introducing immune components derived from human patients into immunocompromised PDX mice. While this partially improves clinical relevance, the murine host environment still limits compatibility of several immune interactions due to species-cross reactivity and may contribute to systemic complications and poor tumor engraftment [151].

Successful engraftment remains a major limitation of the PDX model. Successful engraftment is dependent on preservation of primary tumor viability through minimization of post-resection ischemia prior to engraftment. Immediate (<24 h) subcutaneous engraftment of primary PDAC tumor resection to murine models provided a success rate of up to 66.7% [138,140,152]. Orthotopic xenografts showed similar engraftment rates at 59%, but increased with every new passage up to 90% by the third generation [141], likely due to a shift from the patient-derived stroma to a murine-derived one.

Interestingly, failure to engraft into murine models was associated with better median disease-free survival (DFS: 12.2 months) in their counterpart donor patients compared to successful engraftment groups (DFS: 6.2 months), which highlights a potential marker of recurrence post-surgery [139]. Failed xenografts predicted approximately 81% reduced risk of death in PDAC patients, and this is related to the expression of the tumor-suppressing *SMAD4* gene in failed xenografts. Successful xenografts presented less frequent Smad4 protein, resulting in higher metastatic potential and worse median overall survival (OS: 299 days) compared to failed ones (OS: >800 days) [149]. The correlation of patient outcome with engraftment success was independent of clinical factors like lymph metastasis and size of the primary tumor being engrafted, suggesting that engraftment success may depend on internal characteristics of the primary tumor, such as the viability of tumor cells, the ratio of tumor and stromal cells [139,149,153], and the genetic heterogeneity among individuals.

Collectively, GEMMs and PDXs offer complementary advantages in PDAC research (Table 1). GEMMs are better suited for investigating tumor initiation and progression and, due to their intact immune systems, intrinsic immune interactions. PDXs more effectively preserve patient-specific tumor heterogeneity and clinical characteristics, making them better suited to investigating intratumoral dynamics, drug delivery and efficacy. However, both models remain limited by long establishment times, restricted scalability, and incomplete representation of the intratumoral microbiome. PDXs, in particular, present distinct technical and biological constraints such as the absence of a functional immune system, variable engraftment success, inciting extensive costs and human stroma replacement with murine elements over time.

Overall, the selective addition of microbes in murine models to replicate the intratumoral microbiome is limited to gut microbes, which are not fully translocated to the pancreas and are not representative of the intratumoral microflora. To date, only a single study compared intratumoral microbial signatures between human tissue samples and PDX tissue [150]; therefore, a significant gap remains, as matched patient tumors and their corresponding PDX counterparts have not been comprehensively analyzed. To address this, future research must use 16S rRNA or shotgun sequencing to determine whether progressive microbiome loss or compositional shifts occur following xenograft implantation in PDAC. For mechanistic studies, 3D models can be applied to query the role of specific species on immune infiltration and function, but cannot fully replace the fully integrated data that comes from animal models. While these models can connect the immune-gut-pancreas axis, including stroma [154,155], there are potentially unknown factors whose absence could limit the translatability of the findings.

A majority of current microbial therapeutic strategies (Section 3.3) rely on animal models to understand their mechanisms of action. While these systems provide a valuable preliminary platform for studying MTI interactions, as seen in GEMMs, both human- and bacterial-dependent differences in histology and TME architecture may result in inaccurate representation of human PDAC biology and treatment and ultimately contribute to poor clinical translation of therapeutic outcomes. For instance, Thomas et al. (2018) [74] confirmed the presence of intratumoral microbiota in benign and malignant human surgical resections of PDAC using 16S rRNA sequencing and culture. However, the murine specimens used in this study exhibited intratumoral microbiota in only 50% of transgenic *Kras^G12D^;PTEN^lox/+^* mice and were devoid of intratumoral microbiota in Nod-SCID xenograft mice and gnotobiotic mice. Gnotobiotic mice housed in sterile conditions also did not acquire intrapancreatic microbial populations over time. These observed inconsistencies between complex clinical features in humans and murine models limit the translational capabilities of animal models to human in vivo conditions [74].

To investigate the role of the gut microbiome on PDAC survival, FMT of microbiota derived from human LTS PDAC patients, supplemented to antibiotic-treated murine models, reduced tumorigenesis and increased effective cytotoxic CD8^+^ T-cell infiltration within tumors, compared to STS [77]. However, the immunological and tumor-related effects of FMT are highly dependent on the experimental procedure and donor origin. Specifically, whether the transplanted microbiota is derived from human or murine donors significantly influences immune reconstitution and tumor suppression in germ-free murine models. For instance, Chung et al. (2012) [129] noted that transfer of human- or rat-derived microbiota into gnotobiotic mice failed to fully restore murine immune characteristics, resulting in reduced CD4^+^ and CD8^+^ T-cell populations, diminished dendritic cell numbers, impaired T-cell expansion, and lower production of antimicrobial peptides by intestinal epithelial cells, as compared to murine microbiota. In contrast, supplementation with segmented filamentous bacteria and murine-derived cecal or fecal contents restored T-cell populations and immune maturation [129]. These findings suggest that although human-derived FMT can elicit partial immune responses in murine models, species-specific differences within the microbiota–tumor–immune axis limit the ability of these systems to faithfully recapitulate human immune responses and tumorigenesis.

The continued use of animal models raises not only concerns regarding clinical translatability but also ethical and regulatory considerations. Increasing recognition of the translational limitations of animal models in diseases such as cancer and Alzheimer’s disease has prompted initiatives by organizations such as the National Institutes of Health to encourage a transition toward more human-relevant systems [156]. Collectively, this shift suggests an increasing movement toward reducing reliance on animal models in future biomedical research. To address these shortcomings, 3D culture models, especially spheroids and organoids, have gained prominence. These models strike a balance between the reductionism of 2D culture and the multifaceted integration of human stroma, immune cells and the microbiome seen within in vivo systems, enabling more physiologically relevant yet experimentally tractable studies of PDAC biology.

### 4.2. Three-Dimensional Models: Spheroids and Organoids

3D models have partially recapitulated the complex architecture of both the tumor and its microenvironment while maintaining cell type heterogeneity along with key genotypic and phenotypic traits. In this section, we discuss these 3D models, a term we use here to refer to tumor spheroids or organoids, tissue-based 3D bioprinting, air-liquid interface and microfluidic organ-on-a-chip culture models (Figure 3).

Spheroids are formed by the aggregation of cells from established cell lines in a scaffold-free environment, either through self-assembly or forced culture from single-cell suspensions without the use of ECMs. Heterospheroids are spheroid models composed of multiple cell types, enabling more complex cellular interactions and improved representation of the PDAC TME. Organoids, sometimes referred to as tumoroids [157], are three-dimensional structures derived from primary patient tissues, embryonic stem cells, or induced pluripotent stem cells (IPSCs), and are cultured within artificial extracellular matrices such as Matrigel or collagen to support self-organization and replicate key in vivo features [158]. By preserving cell–cell and cell–ECM interactions, organoid systems overcome limitations of 2D monolayers and tumor cells embedded in ECM gels, more faithfully recapitulating tumor histology, differential responses to chemotherapy, and intratumoral genetic and phenotypic heterogeneity. Patient-derived organoids (PDOs) maintain the patient-specific genetic profile and phenotypic heterogeneity of the primary tumor, providing a platform for investigating personalized medicine.

A major limitation of simple organoids is the absence of key stromal, vascular, metabolic, and immune components of the TME. These elements can be incorporated through co-culture and heterotypic approaches, generating a more physiologically relevant and immune-reactive platform for screening bacterial metabolites [132,159]. For instance, Sharpe et al. (2024) [159] developed a heterotypic system (assembloids) that enabled direct physical interactions between patient-derived esophageal adenocarcinoma organoids and stromal CAFs. This system preserved key features of the primary tumor, including histology, differentiation, and tumor–stroma organization [159]. In a murine breast cancer organoid–immune cell co-culture system, Bacteria-derived butyrate, as discussed above, significantly enhanced ICI-induced tumor cell apoptosis and upregulated CD8 mRNA and IFN-y immune expression, underscoring the systemic relevance of host–microbiome interactions in shaping immunotherapy responses [132].

Another key feature of tumors is the expression of mutant alleles, which can be lost in 2D cultures but maintained in 3D systems. For instance, the prostate cancer mutations SPOP and FOXA1 were not expressed in 2D cell lines, whereas organoids expressed these critical mutations, alongside mutations in genomic instability regulatory genes, chromatin-associated enzymes, and tumor suppressor genes [160,161].

3D PDAC models have provided a reproducible and structurally representative alternative to 2D and animal models, and although there are several advantages to their use, the validity of these models being translational to clinical responders and recapitulating PDAC in an experimental setup needs further development. On their own, spheroids and organoids lack many components of the physiological TME, such as fibroblasts, immune and endothelial cells [162]. The co-involvement of stromal cells and immune cells with spheroids or organoids may fill the gaps in these models, providing a valuable platform for advancing both basic and translational research in immunotherapy for advanced-stage cancers [163].

One major advantage of incorporating stromal and immune components into 3D culture systems is the improved ability to recapitulate the dense and fibrotic PDAC microenvironment observed clinically, a defining feature of PDAC that strongly influences disease behavior and therapeutic response (Figure 1). Durymanov et al. (2019) [13] developed 3D in vitro microtumors as a heterospheroid model with a combination of the PDAC cell line PANC-1 and the murine fibroblast NIH-3T3 cell line. The combined heterospheroid model exhibited improved ECM production in the microtumors, including the presence of fibronectin, fibrillar collagen I, laminin, and hyaluronan [13], components representative of clinical PDAC relevance. The dense stroma prevented nanoparticle penetration, mimicking the impaired influx of interstitial fluid towards lymphatic capillaries as observed in clinical PDAC tumors and murine xenograft models [164]. Spheroids containing only PANC-1 lacked fibronectin production and, in extension, were unable to produce fibrillar collagen I network as exhibited in the heterospheroid model.

In vivo hypoxic conditions inhibit CD8^+^ T-cell function and promote an immunosuppressive TME, contributing to the PDAC’s poor response, particularly to immunotherapies. Hypoxia stimulates quiescent fibroblasts into α-SMA-expressing myCAFs in vivo, the subcutaneous injection of two-week-old heterospheroids with pre-existing hypoxic cores into murine models recapitulated the hyperplasia seen in the ductal epithelium of PDAC more closely. Additionally, the heterospheroids showed lower caspase activity after two weeks of growth compared to PANC-1 only homospheroids, underscoring the pro-survival effect of fibroblasts on tumorigenicity [13]. In extension, Pednekar et al. (2021) [4] employed a unique method to mimic the spatial arrangement and dense fibrotic stroma of PDAC, termed µtissues, by encapsulating a spheroid of PANC-1 cancer cells in a collagen hydrogel embedded with PSCs. This model effectively recapitulated the desmoplastic architecture characteristic of PDAC, demonstrating a PDAC-relevant gene expression profile (upregulated COL1α1, POSTN, FN1, MMP2, αSMA, VCL, PDGFRβ, TGFβR, and VIM genes) comparable to transcriptomic patient data and facilitating the evaluation of anti-fibrotic therapies [4]. The heterogeneous heterospheroid culture and layered µtissue model are illustrated in Figure 3.

Heterospheroid systems have also supported immunological investigations in gastric, CRC, and PDAC models through the incorporation of immune components such as PBMCs isolated from healthy donor blood [32,158,165]. However, they have several limitations. While PBMCs that infiltrate into the heterospheroid likely undergo some amount of education, they will not fully reproduce the native tumor immune infiltrate and can introduce human leukocyte antigen (HLA)-mismatch and immune-mediated tumor cell rejection [153]. Additionally, while heterospheroids provide useful baseline platforms for exhaustive mechanistic testing, the generation of spheroids is commonly dependent on commercial immortalized cell lines or murine clonal cells and is therefore unable to replicate patient-specific heterogeneities. Patient-derived tissues are not typically used in spheroid systems, as they lack the supporting ECM and stromal complexities required to maintain patient-specific features.

Using organoids in place of spheroids, on the other hand, can further expand on architectural complexity while preserving parent tumor characteristics and avoiding HLA-associated cell rejection. PDOs established from resected tumors or fine-needle biopsies [137] (Figure 3), have been validated as predictive tools for chemotherapy sensitivity, and systematic manipulation of TME factors, such as investigating the effects of bacterial metabolites on ICI efficacy [72,132]. Pancreatic progenitor-organoids differentiated from human pluripotent stem cells retained the original phenotypic heterogeneity, histoarchitecture, and patient-specific epigenetic landscapes of the parental tumor. Importantly, they retained the hypoxic adaptation and variable response to targeted therapies as observed in the patient [166].

Aside from recapitulating the PDAC physiology and its TME, incorporating immune components into 3D culture models is important for understanding immunological mechanisms contributing to clinical therapy resistance. Co-culture systems are multicellular in vitro models that combine PDAC tumor cells with stromal and immune components to better recapitulate the native TME [72]. They have the potential to be valuable for the assessment of immunotherapeutics such as ICIs in the context of T-cell infiltration [167] as well as drug screening [166].

The utility of complex organoids using autologous components has been demonstrated outside of PDAC. For instance, Xie et al. (2025) [102] established patient-derived colorectal cancer organoids incorporating matched CAFs and immune cells from the same patients, generating a more representative immunosuppressive TME model. Using this system, the authors demonstrated that the probiotic *C. butyricum* enhanced anti-PD1 efficacy within the co-culture platform. In agreement with translational capability, *C. butyricum* fecal populations positively correlate with patients who respond to ICI therapy [102]. While this study is promising, the results cannot be directly translated to PDAC without further investigation.

In PDAC, several studies have co-cultured PDOs with matched CAFs and autologous or allogenic immune cells [27,102,167]. Tsai et al. (2018) [167] found that PDAC organoids formed from patient-derived tissue closely recapitulate in situ tumors compared to organoids formed from cell lines or xenografts. The same study showed that the addition of T-cells to organoid media external to empty Matrigel domes exhibited no T-cell infiltration; all T-cells lined the perimeter of the dome. However, upon the addition of PDAC PDOs containing matched CAFs to the Matrigel domes, the T-cells penetrated the Matrigel domes and infiltrated the PDOs [167]. In support of adding CAFs to 3D models, Gorchs et al. (2019) [27] used both allogeneic and autologous immune cells with patient-matched CAFs isolated from PDAC resections and found similar immune reactions in both types of immune cells. This is an exciting finding, as it might open up the opportunity for the use of non-matched PBMCs in models, making it easier to establish long-term models. It is important to note, however, that this study was not conducted in a PDO model but in a transwell setup. The study also identified that CAFs and PGE2 induced the expression of co-inhibitory receptors on stimulated T-cells, such as CTLA-4, LAG-3, TIM-3, and PD-1. The expression of PD-1 and co-expression of PD-1 and TIM-3 in the presence of matched CAFs were negatively associated with CD8^+^ T-cell proliferation. Furthermore, after being exposed to CAFs, T-cells that expressed the inhibitory molecules produced lower levels of IFN-y, CD107a and TNF-α. Blocking the immune checkpoint receptors PD-L1 and PD-L2 present on CAFs resulted in restoration of CD8^+^ and CD4^+^ T-cell proliferation. When CAFs were added to unstimulated PBMCs, the proportion of CD4^+^FOXP3^+^ T_regs_ significantly increased. While these findings provide support for incorporating CAFs into 3D PDAC models, they were obtained using a transwell co-culture system rather than a PDO model [27].

Collectively, these findings suggest that using PDOs cultured with immune and stromal cells allows CAFs to induce T-cell exhaustion and immunosuppressive T-cell expression, and prevent cytotoxic T-cell infiltration, all of which are attributable to clinical PDAC and thus show translation capabilities. Regardless of the presence or absence of immune cells, several matched organoid-CAF co-culture models also demonstrated pro-tumorigenic inflammatory and EMT-related gene expression, and increased resistance to chemotherapeutic drugs [167,168,169].

Another 3D system involving patient-derived models, stromal cells and incorporated immune cells is the Interaction with Organoid-in-Matrix (InterOMax) model established by Lahusen et al. (2024) [54]. The InterOMax model consists of murine KPC clonal PDAC spheroids or human PDAC PDOs with stromal primary PSCs, which are inactivated CAFs, and isolated PBMCs from healthy donors on an agarose microwell chip array system (Figure 3). The PDOs in this platform preserved patient-specific heterogeneity within each organoid, while enabling investigation of the molecular mechanisms underlying T-cell responses within the PDAC TME. Organoids present in the center of commonly used scaffold or matrix domes (i.e., Matrigel matrix) are not equally exposed to T-cells as compared to organoids on the outer edges of the dome. In contrast to regular matrices, the uniform PDOs within the InterOMax system were evenly spaced and had a thin and flattened matrix layer, allowing a consistent rate of T-cell penetration into the PDOs. In the presence of PSCs, this model demonstrated an increase in FOXP3 marker for T_regs_ and a decrease in T-cell activation markers (IL-2, IFN-y, granzyme B). These observations signify that the system is representative of PSCs’ clinical PDAC features. The limitations for this model included poor sensitivity of immunofluorescent staining of infiltrated T-cells, reliance on endpoint rather than real-time infiltration detection, a collagen-only matrix not fully representative of patient ECM complexity, and the use of unmatched source of T-cells from PBMC donors [54].

Microphysiological gradients are missing from many static 3D models. To address this omission, some researchers have incorporated microfluidics into their organoid-based models. Microfluidic devices contain microscopic channels, with multiple dimensions under 1 mm, that direct controlled microfluid flow through separate compartments housing different cell populations. This allows manipulation of individual compartments under defined conditions. The defined microchannel architecture and controlled fluid flow improve organoid homogeneity and allow tighter control over culture conditions, while laminar flow enables the establishment of soluble factor gradients across the system. Droplet emulsion microfluidics support reproducible micro-organoid formation from low-volume patient samples [170,171,172]. Microfluidic platforms have been used to investigate a wide range of cancer-related processes, including tumor cell migration, inflammatory cytokine-driven metastasis, ICI responses, and broader TME interactions across multiple cancer types, including melanoma, lung, breast, prostate, lymphatic, and pancreatic cancers [171].

Haque et al. (2022) [173] developed an organoid system with microfluidic tumor-on-a-chip devices that incorporate the desmoplastic-initiating PSCs and macrophages to effectively recapitulate the desmoplastic and immune-rich milieu of PDAC (Figure 3). This advanced platform not only preserves cellular function and longevity but also enables the evaluation of therapeutic efficacy in a patient-specific manner, providing a more accurate representation of patient drug responses and enhancing the translatability of preclinical findings [173]. Tumor-on-a-chip models offer a highly customizable platform for specific experimental needs, but this flexibility also comes with trade-offs, as each new investigation often requires redesign and optimization of the system. As a result, these models can be resource-intensive and time-consuming to establish and adapt, particularly when adjusting configurations for different biological questions.

Both the tumor-on-a-chip and InterOMax models demonstrate the ability of advanced 3D in vitro systems to integrate tumor, stromal, and immune components while maintaining key PDAC features such as cell–cell and cell–matrix interactions and patient-specific intratumoral heterogeneity. These models provide more representative platforms for investigating immunotherapy responses and evaluating patient-specific drug responses with newer generations of 3D systems, progressively addressing limitations identified in earlier models.

PDOs can be used to assess frontline treatment efficacy and identify new treatment sensitivities that emerge after chemotherapy or during tumor relapse, helping guide personalized second-line treatment strategies [72,168,174]. Supporting their translational utility, PDOs established from PDAC patient biopsies have demonstrated chemotherapeutic drug-sensitivity profiles that correlate with corresponding patient treatment outcomes, highlighting their potential as predictive platforms for precision medicine. Importantly, marked inter-patient variability in drug sensitivity was observed, with individual PDOs exhibiting distinct responses to both chemotherapy regimens and their constituent agents. Translation of these dose–response profiles into predictions of clinical disease control further supports the potential of PDOs to inform patient-specific treatment strategies. Importantly, patient-specific drug-response profiles could be generated within a timeframe compatible with clinical decision-making. This supports the feasibility of integrating PDO-guided treatment selection into routine oncology practice, allowing for refinement of therapeutic strategies prior to decisions regarding treatment continuation or alternative interventions that are required. This study, however, reported difficulties in establishing PDOs suitable for drug evaluation from the limited biopsy samples and suggested that larger clinical samples are required to further validate these findings [175]. In CRC, PDO response to chemotherapeutic drugs oxaliplatin and 5-FU was positively correlated with the corresponding patient drug response, displaying heterogeneity in treatment response and drug sensitivity, despite limited biopsy samples. Categorizing PDOs as sensitive and resistant provided clinically translatable responses in patient outcomes. Additionally, PDOs mirrored treatment resistance observed in patients with prior 5-FU exposure, further supporting their value as representative models of clinical drug response. These findings highlight the importance of collecting biopsy samples before treatment to preserve baseline tumor characteristics and drug sensitivity. This study also critically evaluated and suggested protocol optimizations, including media components, readout types and drug concentrations that may lead to more standardized practices for the use of PDOs in personalized medicine [176].

Nevertheless, PDOs still have notable limitations. One of the main challenges associated with organoid models is the lack of standardization across studies. Variations in ECM selection, organoid formation protocols, and culture conditions can all influence organoid morphology and behavior. Achieving uniform organoid size is also difficult, as multiple organoids of varying diameters often develop within the same matrix, resulting in inconsistent cell-to-extracellular fluid ratios. There are differences in growth media components, leading to alteration of the transcriptional landscape and biases in the representation of specific tumor subtypes. PDOs’ dependence on growth factors may also apply selective pressure that enriches for organoids carrying PDAC driver mutations [72]. These challenges highlight the need for models that more accurately capture tumor heterogeneity and TME complexity.

Increasing evidence continues to uncover the influence of the microbiota, immune subpopulations, and stromal components on PDAC progression and tumorigenicity, yet a fully comprehensive model integrating all these factors has not yet been established. The primary gap is the lack of standardized protocols for co-culturing PDOs with bacterial cultures, which complicates reproducibility and inter-study comparisons [12,72,177]. Furthermore, most existing platforms struggle to accommodate anaerobic species or to preserve spatially protected microbial niches, both of which are central to host–microbe dynamics in PDAC. Ongoing optimization of co-culture conditions and differentiation protocols will be essential for incorporating immune and microbial compartments into organoid models. Beyond successful incorporation of bacteria, these models must demonstrate host cell viability, as well as microbial viability and persistence within the TME. Functional immune responses and host–microbe interactions should be confirmed through cytokine analysis, immune-cell activation, and assessment of tumor-cell responses. Phenotypic changes can be evaluated by flow cytometry and microscopy, while alterations in gene expression can be measured using qPCR or RNA sequencing, ideally using single-cell and timecourse experiments to maintain the spatial and temporal subtleties of the PDAC TME. The development of such heterotypic co-cultures will be critical for advancing mechanistic studies and therapeutic discovery in PDAC.

### 4.3. Heterotypic 3D Models in PDAC and Other Cancers

As 3D models become more refined, an emerging frontier lies in their use to interrogate how microbial interactions shape tumor biology and therapeutic outcomes in PDAC. While tumor–stroma and tumor–immune PDAC co-cultures have been established, the deliberate integration of microbes into these systems remains limited. Dissecting microbial effects on tumor immunity has been difficult ex vivo, often forcing reliance on in vivo murine models, where direct interactions are challenging to analyze due to both the complexity of the system and the lack of taxonomic overlap with human microbiomes [72]. Incorporating microbes into immune-reactive organoids provides a tractable system to study these dynamics in a physiologically relevant context. These heterotypic integrative models enable exploration of how tumor-resident microbes shape immune surveillance and, in extension, modulate responses to immunotherapy (Figure 3).

Heterotypic culture models incorporating tumor, stromal, immune, and microbial components are becoming increasingly used in CRC and intestinal research. Heterotypic systems of varying complexities have been developed, ranging from direct supplementation of microbial and immune components into organoid media to localized targeted approaches such as microinjection, and more advanced vertically compartmentalized tumor-on-a-chip platforms. While the majority of proof-of-concept studies have been performed using gut microbes on intestinal platforms, all of the above-mentioned approaches hold potential for incorporating intratumoral microbiota into PDAC in vitro investigations.

Starting with the simplest approach, direct addition of immune components and microbes to immune cells or organoid media has been widely used to investigate the influence of specific intratumoral microbes on ICI therapy response. In a CRC investigation, PDOs and autologous TILs were co-cultured to form the in vitro heterotypic culture system. Autologous TILs were exposed to the selected probiotic, *Clostridium butyricum*, in addition to human anti-PD1 or IgG supplements, prior to addition to the PDOs. *C. butyricum* suppressed IL-6 secretion and infiltration of TAMs, while consequently activating CD8^+^ T-cells, altering the immunosuppressive nature of CRC and improving anti-PD1 effectiveness [102]. Similarly, the immune-reactive tumor organoid (iTO) model developed by Shelkey et al. (2022) [132] combined murine tumor-derived breast cancer organoids with matched splenocytes and showed that bacterial metabolites enhanced ICI-induced tumor cell apoptosis, increased CD8^+^ T-cell survival, and upregulated IFN-γ and CD8 mRNA expression. Together, these studies demonstrate that these models can be used to understand how microbiota and their metabolites can directly modulate immune responses and improve immunotherapy outcomes across different tumor systems (Figure 3).

In order to upgrade any of these models to include a microbial component, technical obstacles must be overcome. The addition of microbes via media external to the organoid requires the bacteria to first infiltrate the organoid structure. This is not a uniform or robust process and can only be done using aerotolerant bacteria. On the other hand, the more targeted approach of microinjection bypasses this step by introducing them directly into a defined niche that better reflects their native environment; however, it is technically challenging, inconsistent, and the ratio of bacteria to human cells cannot be precisely controlled [178].

Microbial microinjection is a favorable technique to consider for co-culturing anaerobic species with solid tumors like PDAC, as it directly introduces the target microbes into the hypoxic luminal centers of the tumor organoid. In PDAC, this is particularly relevant as intratumoral bacteria are likely to localize within hypoxic tumor cores in situ. Microbial agents can be microinjected into the center of intact organoids, mirroring in vivo infections while maintaining human specificity. Developing non-cancerous intestinal organoids from human IPSCs allowed for patient-specific rapid recreation of host genetic variation within the models. Microinjection of human IPSC-derived organoids with commensal and pathogenic strains such as *Salmonella enterica* serovar Typhimurium [179] and Shiga toxin-producing O157:H7 *E. coli* strain [180] induced cytokine production, innate immune activation, and broader transcriptional changes. Imaging further demonstrated epithelial barrier invasion, with *S.* Typhimurium localizing within *Salmonella*-containing vacuoles, while invasion-deficient *invA* mutants showed reduced epithelial penetration as would be expected. Furthermore, fluorescently labeled polymorphonuclear leukocytes introduced into the surrounding media allowed visualization of immune infiltration, showing accumulation at the organoid perimeter, migration through the tissue, and eventual localization within the lumen [179,180].

While PDOs provide strong architectural relevance and allow interaction between tumor, stroma, and immune components, they offer limited control over spatial compartmentalization and microenvironmental gradients. In this context, heterotypic tumor-on-a-chip systems enable more precise modeling of the tumor–microbe axis within defined compartments. For instance, the intestine naturally exhibits distinct luminal and basal vascular regions with corresponding oxygen gradients, a feature that is also characteristic of the hypoxic and heterogeneous PDAC microenvironment. Reflecting this, the microbiota–intestine-on-a-chip model developed by De Gregorio et al. (2022) [181] incorporated a 3D human intestinal organoid of Caco-2 epithelial cells and intestinal myofibroblasts within a porous gelatin scaffold, with commensal bacteria (*Lactobacillus rhamnosus* and *Bifidobacterium longum*) maintained in an anaerobic luminal chamber and PBMCs positioned in the basal compartment. This arrangement maintained a luminal–basal oxygen gradient and enabled vertical stratification of microaerophilic and obligate anaerobic bacteria, closely replicating the spatial and microbial organization of the intestinal environment, while helping elucidate the protective roles of the microbiota against epithelial injury and inflammation [181]. Adaptation of similar compartmentalized chip-based systems for PDAC research may provide a valuable approach for replicating hallmark features of the disease, including collapsed vasculature and hypoxic tumor cores generated through center-focused oxygen gradients. Such models could allow clearer investigation of oxygen-dependent cellular interactions within the PDAC TME. Dedicated microbial compartments may additionally provide controlled environments for culturing and introducing PDAC intratumoral microbes, potentially isolated directly from patient tumor samples, into different experimental configurations depending on the investigation.

While several studies have shown promising results using 3D models for interrogating the microbiome-tumor-immune axis, similar approaches are extremely limited in PDAC. Tajpara et al. (2025) [14] focused on bacteria isolated from pancreatic intraductal papillary mucinous neoplasms (IPMNs) and established a cross-culture system involving heterospheroids and immune cells. 3D heterospheroids made of PANC-1 cells and murine PSCs were co-incubated with IPMN-derived bacteria prior to the addition of human mucosal-associated invariant T (MAIT) cells (Figure 3). MAIT cells can identify IPMN-associated patient-isolated bacteria even in a 3D spheroid environment. Activation of MAIT cells was characterized by increased cytokine production, upregulation of the lymphocyte activation marker CD69 and concomitant TCR downregulation. The creation of the 3D heterospheroid model not only enabled the establishment of physiologically relevant hypoxic conditions but also allowed visualization of bacterial behavior in a tumor-like microenvironment. Notably, *Enterobacter cloacae* and *Enterococcus faecalis* exhibited enhanced pathogenicity under hypoxia. Additionally, bacterial species (*Granulicatella adiacens*, *E. cloacae*, and *E. faecalis*) isolated from IPMN patients with high-grade dysplasia exhibited greater invasive potential into pancreatic spheroids compared to *E. cloacae* and *E. faecalis* isolated from low-grade dysplasia tumors. This observation in particular shows the complex effect of heterogeneity on microbial function within the TME. Collectively, these findings demonstrate that patient-derived intratumoral bacteria from IPMN exhibit context-dependent pathogenicity, disrupting pancreatic cell metabolism and inducing onco-metabolic changes in 3D spheroid models while remaining recognizable to MAIT cells, which respond with immune activation. This highlights the metabolic reprogramming potential of tumor-resident microbes and their pathways as possible therapeutic vulnerabilities in pancreatic tumor biology [14]. It is encouraging to see functional PDAC heterotypic 3D systems that can capture MTI interactions under hypoxic conditions and support appropriate immune recognition. It is important to note, however, that this model does not incorporate PDOs and therefore lacks some of the complexity and clinical specificity of PDAC. Nevertheless, it still represents an important confirmatory step in validating microbe–tumor–immune interactions in PDAC within a controlled 3D system.

Most current studies still rely on animal or 3D models that capture only parts of the system, either PDAC with microbes or PDAC with immune components, rather than fully encompassing the complete MTI axis. Although Han et al. (2024) [93] established a heterospheroid model containing PDAC tumor cells and PSCs to test the infiltration and antibacterial capabilities of the *Lactobacillus rhamnosus* GG probiotic, the immunological cross-study was carried out in mouse models that were subcutaneously and orthotopically injected with PDAC cell lines. Similarly, other research [13,77] used murine approaches, thereby foregoing the mechanistic precision and physiological relevance that 3D in vitro models can offer on the microbe-immune-tumor axis [181]. Despite the successful development of multifactorial 3D models in CRC and gastric cancer, similar systems remain limited in PDAC, likely due to the challenges behind reconstructing its highly complex stromal, fibroblast, and immune microenvironment in an in vitro setup. While these interactions are inherently present in murine models, their ability to faithfully represent human PDAC remains limited. On the other hand, established transwell and cancer-on-a-chip models in CRC and gastric cancer have provided a robust platform for studying microbe–tumor–immune interactions, whereas comparable PDAC models remain underdeveloped. Consequently, establishing a robust and representative PDAC model is a necessary first step prior to incorporating additional factors such as the microbiome and its effects on the TME.

In a recently published standardized protocol, Tajpara et al. (2025) [14] demonstrate the incorporation of immune cells into heterospheroids containing the immortalized PDAC cell line PANC-1 and CAFs. The model represents a crucial first step toward pancreatic 3D models that capture the multidirectional interactions between tumor cells, immune populations, and microbes. Briefly, PANC-1/CAF heterospheroids were grown for 8 days at 37 °C and 5% CO_2,_ wherein the option of adding PBMCs extracted from healthy individuals can be added to the heterospheroids at Day 4. While several attempts have been made thus far towards the formation of heterospheroids [4,13,14,93], they lacked the standardization required for interpretation across studies. This newly validated and tested protocol is a favorable advance towards standardized immune-reactive 3D models to investigate microbial intervention in PDAC. The consideration of adding bacterial components to this standardized multicellular model could be a potential solution for investigating cross-interactions. Co-culturing bacterial strains with isolated patient PBMCs was previously carried out to investigate the influence of live, heat-killed and supernatant bacterial fractions on immune responses [79,182]. The addition of these induced bacteria-immune populations could potentially be added into 3D heterotypic models to study the MTI axis as a whole.

While there is precedent in establishing a microbially inclusive and immune-reactive 3D PDAC model, no system comes without limitations. The establishment of physiologically representative heterotypic systems remains constrained by challenges associated with standardization, scalability, reproducibility, and the integration of multiple biological components, with these limitations varying according to the model employed.

During spheroid formation, oxygen gradients are readily established due to diffusion limitations, resulting in hypoxic cores. However, maintaining hypoxic cores is challenging and strongly size-dependent, as spheroids exceeding ≥500 µm often develop diffusion-limited oxygen transport, leading to central anoxia and eventual necrosis [183]. Maintaining a physiologically relevant hypoxic core without extensive necrosis is critical, as excessive cell death may alter the microenvironment and compromise intratumoral microbial and immune viability. Hypoxic chambers can be used to partially recapitulate tumor hypoxia in vitro, while emerging 3D culture platforms, including bioprinting, bioreactors, and microfluidic systems, provide more refined control over oxygen gradients and nutrient availability, facilitating more physiologically relevant modeling of the TME [184].

Upon establishing hypoxic cores in 3D PDAC heterospheroid and organoid models, the presence of oxygen gradients within the model necessitates careful consideration of both bacterial species selection and site of introduction. For instance, bacteria microinjected into the hypoxic core would likely need to be obligate or facultative anaerobes. Most microbial associations with PDAC patient outcomes have been reported at higher taxonomic levels (Supplemental Table S1), necessitating the identification and validation of causal species within physiologically relevant heterotypic systems. Such models should also demonstrate preservation of biologically relevant microbial functions, including metabolite production, immune modulation, and virulence-associated phenotypes, while accurately recapitulating host–microbe interactions within the TME. Although bacterial incorporation into 3D culture systems has been successfully achieved for short-term studies, maintaining long-term microbial persistence without compromising cellular viability, functionality, or structural integrity remains challenging [185,186]. Excessive bacterial expansion may disrupt tissue architecture and reduce cell viability, whereas insufficient colonization may fail to reproduce physiologically relevant microbial effects. Furthermore, experimental outcomes can be substantially influenced by inoculation parameters, including the route, timing, and multiplicity of infection, highlighting the importance of standardized protocols to improve reproducibility and facilitate inter-study comparisons. Air–liquid interface systems have shown some success in supporting prolonged host–microbe co-culture through the use of reduced inoculation loads, although further optimization is required for chronic disease modeling [186].

In addition to microbial inoculation, the incorporation of immune components introduces additional sources of biological variability and technical complexity. In reference to the standardized protocol by Thomas et al. (2026) [165], additional challenges arise from donor-to-donor variability when using PBMCs isolated from human donor blood alongside immortalized cancer cell lines. Furthermore, the limited availability of donor blood often requires PBMC cryopreservation for future use, which can reduce cytokine production and the proportion of antigen-presenting cells [187], while prolonged storage can significantly reduce cell capture efficiency in single-cell RNA sequencing [188]. Finite donor samples and biological variability can complicate model standardization and reproducibility over time.

Immune compatibility between incorporated cellular components may further influence model reproducibility. Although not commonly reported in spheroid models derived from immortalized cancer cell lines, potential HLA-mediated incompatibility may have significant immune effects in PDO models, posing challenges for reproducibility and standardization when primary immune cells are incorporated. In addition to cellular compatibility, the structural components used to reconstruct the TME represent another important source of variability. Because the microenvironment is reconstructed from the ground up, spheroid and organoid models often heavily rely on exogenous matrices such as collagen [4,165] or Matrigel [168,169] for structural support.

Among these matrices, Matrigel remains the most widely used and consequently warrants particular consideration. Variations in Matrigel stiffness can differentially regulate cellular responses depending on the tissue of origin, thereby changing cell morphology and lineage differentiation [189], supporting tissue-specific experimental design. Although Matrigel is widely regarded as the gold standard for 3D cultures and supports multiple cell types, its murine origin and batch-to-batch variability raise concerns regarding standardization, reproducibility, and translational relevance [189]. In addition, Matrigel can influence drug efficacy in organoid cultures by acting as physical and biochemical barriers that alter drug diffusion and create spatial gradients of media-derived factors within organoid domes, thereby contributing to variability in treatment responses and limiting experimental consistency [190]. In the context of PDAC models, the complex ECM composition of Matrigel, including laminin and collagen IV [189], may further introduce non-physiological stromal cues and exacerbate fibrotic features characteristic of the disease. Although widely used, Matrigel should be carefully selected based on the cancer type and experimental objective, as it does not fully recapitulate patient-specific ECM heterogeneity observed in vivo. By comparison, decellularized ECM from target organs or tissues more faithfully preserves native biochemical composition and biomechanical properties. Alternative ECM, such as silicon-based hydro-microwell plate, showed more homogenous CRC PDO formation, and similar to decellularized ECM from target organs or tissues, exhibited increased drug response sensitivity in bioprinted gastric cancer models when compared to standardized Matrigel cultures, highlighting the importance of cell-ECM interactions in drug prediction [191,192].

The inherent complexity of PDAC heterotypic models presents significant scalability challenges. Simultaneously maintaining tumor, stromal, immune, and microbial compartments, while preserving physiologically relevant hypoxia and host–microbe interactions, requires complex culture systems with stringent environmental control and multi-component coordination, which limits throughput and broader implementation. Integration of advanced 3D models with high-throughput technologies and artificial intelligence-based analytical approaches may help address these scalability challenges [193,194]. For instance, PDOs can present challenges in successfully establishing organoids that are suitable for drug testing from limited biopsy samples [175]. However, implementing PDOs into high-throughput systems like the previously described InterOMax ensures scalability and high reproducibility as their agarose microwells establish multiple uniform organoids, free of added matrix influence, and account for matched stromal and immune components [54]. Collectively, these limitations highlight the need to balance physiological complexity with experimental reproducibility when developing heterotypic PDAC models, while emerging platforms increasingly address many of the constraints associated with conventional 3D culture systems.

PDAC research has been conducted thus far using a range of models from the simplistic 2D monolayer of cells, to animal models and finally to the most representative 3D models of PDAC. Despite major advances, animal models still fail to fully recapitulate the heterogeneity and complex microenvironment of clinical PDAC, contributing to the growing shift toward 3D model systems. 3D PDAC models have been used in various setups incorporating tumor and immune components, and in some cases, probiotic effects on tumor organoids. However, there is a severe lack of initiatives incorporating the MTI axis into 3D models despite their evidenced integrative roles in PDAC tumorigenesis and poor immunotherapeutic response. Developing an appropriate model can open avenues to investigate and further improve immunotherapies.

## 5. Future Considerations for PDAC

Intratumoral and gut microbiota have been shown to directly and indirectly influence the cancer TME and potential responses to immunotherapies in a variety of cancers. In order to more effectively investigate the complex network between the microbiome, immune system, and PDAC tumor, a standardized and representative heterotypic, three-dimensional system is suggested. Approaches for investigating microbe influence on tumorigenesis, immune modulation and therapy resistance in organoid models have been widely and successfully applied in CRC and gastric cancers [12,102,179,180,181]. However, microbial integration into PDAC research has been surprisingly limited despite the availability of replicative 3D models [4,13,93,165]. To date, the only documented attempt of a microbe-inclusive and immune-reactive 3D PDAC system was successfully performed by Tajpara et al. (2025) [14] involving PDAC/fibroblast heterospheroids incorporated with MAIT cells and bacterial species isolated from pancreatic IPMNs. This work demonstrates a clear link between intratumoral microbiota and pathogenesis, and encourages further studies using these types of advanced 3D models. Several studies have confirmed the association of microbes with immune modulation as well as PDAC prognosis and ICI treatment response. However, further research towards tunable heterotypic 3D systems revolving around the MTI axis presents opportunities in identifying causality. 

The lack of existing heterotypic model studies in PDAC presents a major gap in advancing PDAC research and treatment development. The standardized immunoreactive 3D PDAC protocol provided by Thomas et al. (2026) [165] may be the first step towards inciting more microbe-driven PDAC investigations. Expanding such systems could provide valuable insights into the complex immuno-microbial dynamics that shape PDAC tumor biology and therapeutic response. Although 3D models can be more modifiable than 2D models, more affordable than animal models and better representative of disease as compared to both, it is also important to recognize that host–microbe interactions characterized in vitro may not fully mirror those observed in vivo, underscoring the need for contextual validation within physiologically representative models [195]. Information obtained from these inclusive heterotypic models may help identify patient-specific therapeutic vulnerabilities, predict treatment responses, and optimize immunotherapeutic strategies. Furthermore, 3D models enable the controlled introduction of selected microbes to evaluate their potential as probiotic or microbiome-targeted adjuvant therapies, while simultaneously assessing their invasiveness and tumor-promoting capacity. Given that bacterial strains associated with advanced disease can exhibit greater invasive behavior than those isolated from less aggressive lesions [14], such models provide a clinically relevant platform for investigating the functional consequences of microbe–tumor interactions and their therapeutic implications in PDAC.

3D models offer an opportunity to establish directionality and causality for microbe-host interactions in PDAC. With further advances, multi-organ-on-chip systems [196] would allow clinicians to personalize treatments to a patient’s MTI axis prior to administering this treatment to the patient. The advancement of multi-organ-on-chip systems in combination with emerging in silico techniques [197] is likely to vastly improve patient outcomes [198] in PDAC over the coming decade.

## Data Availability

No new data were created or analyzed in this study. Data sharing is not applicable to this article.

## Supplementary Materials

The following supporting information can be downloaded at: https://www.mdpi.com/article/10.3390/nu18132113/s1. Table S1: Summary of patient microbiomes in healthy volunteers and PDAC patients.

## Abbreviations

The following abbreviations are used in this manuscript:

PDAC	Pancreatic ductal adenocarcinoma
PSCs	Pancreatic stellate cells
CAFs	Cancer-associated fibroblasts
ICI	Immune checkpoint inhibitor
PD-1	Programmed cell death 1
PD-L	Programmed cell death ligand 1
CTLA-4	Cytotoxic T Lymphocyte-associated-protein 4
TME	Tumor microenvironment
PDOs	Patient-derived organoids
ECM	Extracellular matrix
IL	Interleukin
FAP	Fibroblast activation protein
FASL	Fas ligand
CD	Cluster of differentiation
TGF-β	Transforming growth factor beta
TNF-α	Tumor necrosis factor alpha
MHC	Major histocompatibility complex
TAMs	Tumor-associated macrophages
GM-CSF	Granulocyte-macrophage colony-stimulating factor
CCR8	C-C motif chemokine receptor 8
CCL	C-C motif chemokine ligand
TILs	Tumor-infiltrating lymphocytes
MDSCs	Myeloid-derived suppressor cells
PGE2	Prostaglandin 2
FOXP3	Forkhead Box Protein 3
MTI	Microbe-tumor-immune
IPMN	Intraductal Papillary Mucinous Neoplasm
T_regs_	Regulatory T-cells
CTLs	Cytotoxic T-lymphocytes
myCAFs	Myofibroblastic CAFs
α-SMA	Alpha-smooth muscle actin
CRC	Colorectal cancer
LPS	Lipopolysaccharide
LTS	Long-term survivors/survival
STS	Short-term survivors/survival
CXCL1	C-X-C motif chemokine ligand 1
MIP-3α	Macrophage Inflammatory Protein-3 alpha
CXCR2	C-X-C motif chemokine receptor 2
TLR-	Toll-like receptor
MyD88	Myeloid differentiation primary response 88
NF-κB	Nuclear factor kappa-light-chain-enhancer of activated B cells
BCR	B-cell receptor
TCR	T-cell receptor
FMT	Fecal microbiota transplantation
secD	Protein translocase subunit SecD
GRP78	Glucose-regulated protein 78
EMT	Epithelial-to-mesenchymal transition
SCFAs	Short-chain fatty acids
MEK	Methyl ethyl ketone
IFN-γ	Interferon- γ
HDAC	Histone deacetylases
AOM	azoxymethane
DSS	Dextran sodium sulfate
SPF	Specific-pathogen-free mice
NSCLC	Non-small cell lung cancer
Th1	T-helper cell-1
iTO	Immune-reactive tumor organoid
GEMMs	Genetically engineered mouse models
PDX	Patient-derived xenografts
DFS	Disease-free survival
OS	Overall survival
LAG-3	Lymphocyte-activation gene 3
TIM-3	T-cell immunoglobulin and mucin domain-containing molecule-3
PBMCs	Peripheral blood mononuclear cells
HLA	Human leukocyte antigens
MAIT	Mucosal-associated invariant T-cells
IPSCs	Induced pluripotent stem cells
