# Supplementary material for: Can Complex 3D Models Effectively Replace 2D and Animal Models to Investigate the Microbe-Tumor-Immune Axis in Pancreatic Cancer Studies?
Source: Nutrients. 2026 Jun 28;18(13):2113. doi: 10.3390/nu18132113 (PMC13362988; doi:10.3390/nu18132113)
Supplement: Supplementary file 1 [file nutrients-18-02113-s001.zip › nutrients-4358465-supplementary.pdf]

Supplemental Table S1: Summary of patient microbiomes in healthy volunteers and PDAC patients.

**PDAC vs. Healthy Controls**								
**Taxa**				**Sample Type**	**Directionality**	**Effect association**	**Limitation**	**Ref**
**Order**	**Family**	**Genus**	**Species**					
Verrucomicrobiales	Akkermansiaceae	*Akkermansia*	-	Fecal samples	Increased	In PDAC patients vs. healthy	Small sample sizes, especially for early-stage groups.<br><br>Single ethnic cohort.	(Half et al., 2019)
Bacteroidales	Odoribacteraceae	*Odoribacter*	-	Fecal samples	Increased	In PDAC patients vs. healthy	Small sample sizes, especially for early-stage groups.<br><br>Single ethnic cohort.	(Half et al., 2019)
	Prevotellaceae	*Prevotella*	-	Fecal samples	Increased	In PDAC patients vs. healthy	Research design unable to account for causality in clinical PDAC. Requires downstream *in vitro* or *in vivo* validation.	(Ren et al., 2017)
			Prevotella pallens	Saliva samples	Increased	In PDAC patients vs. healthy	PDAC sample sizes were small.<br><br>Multiple cohort comparisons, however patient data of antibiotic users for one cohort could not be excluded.	(Nagata et al., 2022)

							Primarily based on shotgun metagenomic sequencing. No *in vitro* or *in vivo* models to confirm causality.	
			Prevotella pallens	Saliva samples	Increased	In PDAC patients vs. healthy	PDAC sample sizes were small.<br><br>Multiple cohort comparisons, however patient data of antibiotic users for one cohort could not be excluded.<br><br>Primarily based on shotgun metagenomic sequencing. No *in vitro* or *in vivo* models to confirm causality.	(Nagata et al., 2022)
		Hallella	-	Fecal samples	Increased	In PDAC patients vs. healthy	Research design unable to account for causality in clinical PDAC. Requires downstream *in vitro* or *in vivo* validation.	(Ren et al., 2017)

Enterobacterales	Enterobacteriaceae	*Enterobacter*	-	Fecal samples	Increased	In PDAC patients vs. healthy	Research design unable to account for causality in clinical PDAC. Requires downstream *in vitro* or *in vivo* validation.	(Ren et al., 2017)
Lactobacillales	Streptococcaceae	*Streptococcus*	*Streptococcus oralis*	Fecal samples	Increased	In PDAC patients vs. healthy	PDAC sample sizes were small.<br><br>Multiple cohort comparisons, however patient data of antibiotic users for one cohort could not be excluded.<br><br>Primarily based on shotgun metagenomic sequencing. No *in vitro* or *in vivo* models to confirm causality.	(Nagata et al., 2022)
			Streptococcus anginosus	Fecal samples	Increased	In PDAC patients vs. healthy	PDAC sample sizes were small.<br><br>Multiple cohort comparisons, however patient data of antibiotic users for one cohort	(Nagata et al., 2022)

							could not be excluded.<br><br>Primarily based on shotgun metagenomic sequencing. No *in vitro* or *in vivo* models to confirm causality.	
			Streptococcus vestibularis	Fecal samples	Increased	In PDAC patients vs. healthy	PDAC sample sizes were small.<br><br>Multiple cohort comparisons, however patient data of antibiotic users for one cohort could not be excluded.<br><br>Primarily based on shotgun metagenomic sequencing. No *in vitro* or *in vivo* models to confirm causality.	(Nagata et al., 2022)
			Streptococcus mitis	Saliva samples	Decreased	In PDAC compared vs. chronic pancreatiti	Small sample size. Lacks participants with smoking history, an	(Farrell et al., 2012)

						s and healthy controls	important PDAC risk factor. Experimental design lacks early-stage analyses and mechanistic insights.	
			Streptococcus salivarius	Surgical resection, 16S rRNA	Decreased	In PDAC tumor compared to adjacent normal PDAC tissues	Participants were restricted to resected PDAC but not metastatic. Lacks causality of intratumoral bacteria towards clinical PDAC pathology. Lacks consideration for confounding factors. Healthy controls lacked age and gender data disallowing relevant categorizing and comparison within demographics.	(Tavano et al., 2025)
				Saliva samples	Decreased	In PDAC patients vs. healthy	PDAC sample sizes were small.<br><br>Multiple cohort comparisons, however patient data of antibiotic users for one cohort	(Nagata et al., 2022)

							could not be excluded.<br><br>Primarily based on shotgun metagenomic sequencing. No *in vitro* or *in vivo* models to confirm causality.	
			Streptococcus thermophilus	Saliva samples	Decreased	In PDAC patients vs. healthy	PDAC sample sizes were small.<br><br>Multiple cohort comparisons, however patient data of antibiotic users for one cohort could not be excluded.<br><br>Primarily based on shotgun metagenomic sequencing. No *in vitro* or *in vivo* models to confirm causality.	(Nagata et al., 2022)
			Streptococcus australis	Saliva samples	Decreased	In PDAC patients vs. healthy	PDAC sample sizes were small.	(Nagata et al., 2022)

							Multiple cohort comparisons, however patient data of antibiotic users for one cohort could not be excluded.<br><br>Primarily based on shotgun metagenomic sequencing. No *in vitro* or *in vivo* models to confirm causality.	
	Carnobacteriaceae	*Granulicatella*	*Granulicatella adiacens*	Saliva samples	Increased	In PDAC tumors compared to both chronic pancreatitis and healthy controls	Small sample size. Lacks participants with smoking history, an important PDAC risk factor. Experimental design lacks early-stage analyses and mechanistic insights.	(Farrell et al., 2012)
Veillonellales	Veillonellaceae	-	-	Fecal samples	Increased	In PDAC patients vs. healthy	Small sample sizes, especially for early-stage groups.<br><br>Single ethnic cohort.	(Half et al., 2019)

		Veillonella	-	Fecal samples	Increased	In PDAC patients vs. healthy	Research design unable to account for causality in clinical PDAC. Requires downstream *in vitro* or *in vivo* validation.	(Ren et al., 2017)
			Veillonella parvula	Fecal samples	Increased	In PDAC patients vs. healthy	PDAC sample sizes were small.<br><br>Multiple cohort comparisons, however patient data of antibiotic users for one cohort could not be excluded.<br><br>Primarily based on shotgun metagenomic sequencing. No *in vitro* or *in vivo* models to confirm causality.	(Nagata et al., 2022)
			Veillonella atypica	Fecal samples	Increased	In PDAC patients vs. healthy	PDAC sample sizes were small.<br><br>Multiple cohort comparisons, however patient data of antibiotic users for one cohort	(Nagata et al., 2022)

							could not be excluded.<br><br>Primarily based on shotgun metagenomic sequencing. No *in vitro* or *in vivo* models to confirm causality.	
Actinomycetales	Actinomycetaceae	*Actinomyces*	-	Fecal samples	Increased	In PDAC patients vs. healthy	PDAC sample sizes were small.<br><br>Multiple cohort comparisons, however patient data of antibiotic users for one cohort could not be excluded.<br><br>Primarily based on shotgun metagenomic sequencing. No *in vitro* or *in vivo* models to confirm causality.	(Nagata et al., 2022)
Bacillales/ Caryophanales	Staphylococcaceae	*Jeotgalicoccus*	-	Surgical resection	Increased	In healthy pancreas vs. tumor and	Participants were restricted to resected PDAC but not metastatic.	(Tavano et al., 2025)

						adjacent normal PDAC tissues	Lacks causality of intratumoral bacteria towards clinical PDAC pathology. Lacks consideration for confounding factors. Healthy controls lacked age and gender data disallowing relevant categorizing and comparison within demographics.	
Moraxellales	Moraxellaceae	*Acinetobacter*	*Acinetobacter guillouiae*	Surgical resection	Increased	In healthy pancreas vs. tumor and adjacent normal PDAC tissues	Participants were restricted to resected PDAC but not metastatic. Lacks causality of intratumoral bacteria towards clinical PDAC pathology. Lacks consideration for confounding factors. Healthy controls lacked age and gender data disallowing relevant categorizing and comparison within demographics.	(Tavano et al., 2025)

Sphingomonadales	Sphingomonadaceae	*Sphingomonas*	-	Surgical resection	Decreased	In PDAC tumor compared to adjacent normal PDAC tissues	Participants were restricted to resected PDAC but not metastatic.<br>Lacks causality of intratumoral bacteria towards clinical PDAC pathology. Lacks consideration for confounding factors. Healthy controls lacked age and gender data disallowing relevant categorizing and comparison within demographics.	(Tavano et al., 2025)
Clostridiales/ Eubacteriales	Clostridiaceae	-	-	Fecal samples	Decreased	In PDAC patients vs. healthy	Small sample sizes, especially for early-stage groups.<br><br>Single ethnic cohort.	(Half et al., 2019)
		Clostridium IV		Fecal samples	Decreased	In PDAC patients vs. healthy	Research design unable to account for causality in clinical PDAC. Requires downstream *in vitro* or *in vivo* validation.	(Ren et al., 2017)
Clostridiales/ Lachnospirales	Lachnospiraceae	-	-	Fecal samples	Decreased	In PDAC patients vs. healthy	Small sample sizes, especially for early-stage groups.	(Half et al., 2019)

							Single ethnic cohort.	
				Fecal samples	Decreased	In PDAC patients vs. healthy	PDAC sample sizes were small.<br><br>Multiple cohort comparisons, however patient data of antibiotic users for one cohort could not be excluded.<br><br>Primarily based on shotgun metagenomic sequencing. No *in vitro* or *in vivo* models to confirm causality.	(Nagata et al., 2022)
		Coprococcus	-	Fecal samples	Decreased	In PDAC patients vs. healthy	Research design unable to account for causality in clinical PDAC. Requires downstream *in vitro* or *in vivo* validation.	(Ren et al., 2017)
		Blautia	-	Fecal samples	Decreased	In PDAC patients vs. healthy	Research design unable to account for causality in clinical PDAC. Requires downstream *in vitro* or *in vivo* validation.	(Ren et al., 2017)

		Anaerostipes	-	Fecal samples	Decreased	In PDAC patients vs. healthy	Research design unable to account for causality in clinical PDAC. Requires downstream *in vitro* or *in vivo* validation.	(Ren et al., 2017)
Clostridiales/ Eubacteriales	Ruminococcaceae/ Oscillospiraceae	-	-	Fecal samples	Decreased	In PDAC patients vs. healthy	Small sample sizes, especially for early-stage groups.<br><br>Single ethnic cohort.	(Half et al., 2019)
		Ruminococcus	*Ruminococcus bicirculans*	Fecal samples	Decreased	In PDAC patients vs. healthy	PDAC sample sizes were small.<br><br>Multiple cohort comparisons, however patient data of antibiotic users for one cohort could not be excluded.<br><br>Primarily based on shotgun metagenomic sequencing. No *in vitro* or *in vivo* models to confirm causality.	(Nagata et al., 2022)
		Faecalibacterium	*Faecalibacterium prausnitzii*	Fecal samples	Decreased	In PDAC patients vs. healthy	PDAC sample sizes were small.	(Nagata et al., 2022)

							Multiple cohort comparisons, however patient data of antibiotic users for one cohort could not be excluded.<br><br>Primarily based on shotgun metagenomic sequencing. No *in vitro* or *in vivo* models to confirm causality.	
		Flavonifractor	-	Fecal samples	Decreased	In PDAC patients vs. healthy	Research design unable to account for causality in clinical PDAC. Requires downstream *in vitro* or *in vivo* validation.	(Ren et al., 2017)
Neisseriales	Neisseriaceae	*Neisseria*	*Neisseria elongata*	Saliva samples	Decreased	In PDAC patients vs. healthy	Small sample size. Lacks participants with smoking history, an important PDAC risk factor. Experimental design lacks early-stage analyses and mechanistic insights.	(Farrell et al., 2012)

**LTS vs. STS**								
Clostridiales	-	-	-	Surgical resection, FFPE sections	Increased	In STS compared to LTS	16S rRNA sequencing was on human samples, but immunocompromised murine PDAC xenografts were used for FMT from human LTS and STS patients, removing immune reaction component and limiting comparability between patient sample and FMT datasets. Human sample-based 3D models may have been a more representative model.	(Riquelme et al., 2019)
	Clostridiaceae	*Clostridium sensu stricto 1*	-	Surgical resection, FFPE sections	Increased	In STS compared to LTS	Lacks causal *in vitro* investigation of LTS/STS associated bacteria and mechanistic investigation of bacterially-enhanced ICI efficacy in murine model. Mice were	(Huang et al., 2022)

							immune competent and could have had reactive immune responses to bacterial inoculation. No confirmatory test conducted on murine tumor to confirm intratumoral presence of bacteria after administering through oral gavage.	
Lysobacterales/ Xanthomonadales	Lysobacteraceae/ Xanthomonadaceae	*Pseudoxanthomonas*	-	Surgical resection, FFPE sections	Increased	In LTS compared to STS	16S rRNA sequencing was on human samples, but immunocompromised murine PDAC xenografts were used for FMT from human LTS and STS patients, removing immune reaction component and limiting comparability between patient sample and FMT datasets. Human sample-based 3D models may have	(Riquelme et al., 2019)

							been a more representative model.	
Pseudonocardiale	Pseudonocardiaceae	*Saccharopolyspora*	*Saccharopolyspora rectivirgula*	Surgical resection, FFPE sections	Increased	In LTS compared to STS	16S rRNA sequencing was on human samples, but immunocompromised murine PDAC xenografts were used for FMT from human LTS and STS patients, removing immune reaction component and limiting comparability between patient sample and FMT datasets. Human sample-based 3D models may have been a more representative model.	(Riquelme et al., 2019)
Kitasatosporales/ Streptomycetales	Streptomycetaceae	*Streptomyces*	-	Surgical resection, FFPE sections	Increased	In LTS compared to STS	16S rRNA sequencing was on human samples, but immunocompromised murine PDAC xenografts were used for FMT from human LTS and STS	(Riquelme et al., 2019)

							patients, removing immune reaction component and limiting comparability between patient sample and FMT datasets. Human sample-based 3D models may have been a more representative model.	
Bacillales/ Caryophanales	Bacillaceae	*Bacillus*	*Bacillus clausii*	Surgical resection, FFPE sections	Increased	In LTS compared to STS	16S rRNA sequencing was on human samples, but immunocompromised murine PDAC xenografts were used for FMT from human LTS and STS patients, removing immune reaction component and limiting comparability between patient sample and FMT datasets. Human sample-based 3D models may have been a more	(Riquelme et al., 2019)

							representative model.	
Veillonellales	Veillonellaceae	*Megasphaera*	-	Surgically resected, FFPE sections	Increased	In LTS compared to STS	Lacks causal *in vitro* investigation of LTS/STS associated bacteria and mechanistic investigation of bacterially-enhanced ICI efficacy in murine model. Mice were immune competent and could have had reactive immune responses to bacterial inoculation. No confirmatory test conducted on murine tumor to confirm intratumoral presence of bacteria after administering through oral gavage.	(Huang et al., 2022)
Sphingomonadales	Sphingomonadaceae	*Sphingomonas*	-	Surgically resected, FFPE sections	Increased	In LTS compared to STS	Lacks causal *in vitro* investigation of LTS/STS associated bacteria and mechanistic investigation of	(Huang et al., 2022)

							bacterially-enhanced ICI efficacy in murine model. Mice were immune competent and could have had reactive immune responses to bacterial inoculation. No confirmatory test conducted on murine tumor to confirm intratumoral presence of bacteria after administering through oral gavage.	
Neisseriales	Neisseriaceae	*Neisseria*	-	Surgically resected, FFPE sections	Increased	In STS compared to LTS	Lacks causal *in vitro* investigation of LTS/STS associated bacteria and mechanistic investigation of bacterially-enhanced ICI efficacy in murine model. Mice were immune competent and could have had reactive immune responses to	(Huang et al., 2022)

							bacterial inoculation. No confirmatory test conducted on murine tumor to confirm intratumoral presence of bacteria after administering through oral gavage.	
Pasteurellales	Pasteurellaceae	*Aggregatibacter*	-	Surgically resected, FFPE sections	Increased	In STS compared to LTS	Lacks causal *in vitro* investigation of LTS/STS associated bacteria and mechanistic investigation of bacterially-enhanced ICI efficacy in murine model. Mice were immune competent and could have had reactive immune responses to bacterial inoculation. No confirmatory test conducted on murine tumor to confirm intratumoral presence of bacteria	(Huang et al., 2022)

							after administering through oral gavage.	
Bacteriodales	Porphyromonadaceae	*Porphyromonas*	-	Surgically resected, FFPE sections	Increased	In STS compared to LTS	Lacks causal *in vitro* investigation of LTS/STS associated bacteria and mechanistic investigation of bacterially-enhanced ICI efficacy in murine model. Mice were immune competent and could have had reactive immune responses to bacterial inoculation. No confirmatory test conducted on murine tumor to confirm intratumoral presence of bacteria after administering through oral gavage.	(Huang et al., 2022)
Lactobacillales	Enterococcaceae	*Enterococcus*	-	Surgically resected, FFPE sections	Increased	In LTS compared to STS	Lacks causal *in vitro* investigation of LTS/STS associated bacteria and mechanistic investigation of	(Huang et al., 2022)

							bacterially-enhanced ICI efficacy in murine model. Mice were immune competent and could have had reactive immune responses to bacterial inoculation. No confirmatory test conducted on murine tumor to confirm intratumoral presence of bacteria after administering through oral gavage.	
